# Native CZE-MS analysis of antibodies and hemoglobin using protein-adapted CZE and ESI conditions for detailed proteoform characterization

**DOI:** 10.1007/s00216-026-06611-1

**Published:** 2026-06-13

**Authors:** Ann-Katrin Schwenzer, Jule Weiß, Sabrina Buntz, Christian Neusüß

**Affiliations:** Faculty of Chemistry, Aalen University, 73430 Aalen, Germany

**Keywords:** Charge variants, Protein complex, Native and denaturing ESI-MS, Adalimumab, Infliximab, Bevacizumab

## Abstract

Capillary zone electrophoresis-mass spectrometry (CZE-MS) under native-like conditions is a selective and efficient tool for studying the proteoforms of proteins or protein complexes. Recently, it has been shown that a neutral capillary coating in combination with an ammonium acetate–based electrolyte with near-neutral pH can result in a high electrophoretic resolution between different proteoforms of an antibody, allowing for detailed characterization of the drug product. Here, we extend the CZE-MS method and demonstrate its flexibility for the analysis of protein constructs having different isoelectric points (pIs) by adapting the pH value of the electrolyte depending on the pI of the protein, the desired separation performance, and analysis time. Depending on the applied sheath liquid composition, the method allows for protein analysis under denaturing or native-like conditions. Several antibodies were analyzed by CZE-MS with a systematic evaluation of the separation of typical charge variants. The high separation performance enables the detection of between 45 (bevacizumab) and 100 proteoforms (adalimumab), including variants not resolvable by MS and proteoforms not reported before. Native CZE-MS analysis of hemoglobin enables the separation of different hemoglobin variants, such as glycated, oxidized, and glutathionylated variants, as well as hemoglobin complexes with different iron oxidation states, while preserving non-covalent interactions of the complex. The alternative application of a denaturing sheath liquid leads to dissociation into monomers, thereby allowing a more reliable assignment of modifications and their localization to the respective subunit.

**Graphical abstract:**

**Figure:**
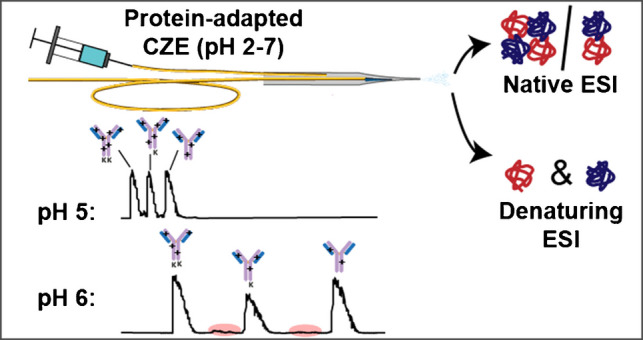

**Supplementary Information:**

The online version contains supplementary material available at 10.1007/s00216-026-06611-1.

## Introduction

Capillary zone electrophoresis (CZE) separates analytes based on the charge and size of the analyte molecule, making it a powerful technique for the separation of proteins and proteoforms that differ in their charge or their higher-order structure. Aqueous background electrolytes (BGEs) such as ammonium acetate (AmAc) can maintain the native conformation of a protein during CZE and enable the analysis of the overall proteoform population of a protein construct [1]. However, the information obtained from stand-alone native CZE is limited, and usually, further techniques are required for the identification and characterization of separated charge variants. Due to recent advances in capillary coating and interfacing, native CZE can be coupled directly to mass spectrometry (MS), enabling the separation of different protein constructs and their direct identification [2].

The analysis of proteoforms by CZE hyphenated to MS is a valuable tool for characterizing therapeutic proteins, as well as endogenous proteins and protein complexes [3–6]. Monitoring of therapeutic proteins, such as therapeutic antibodies, is crucial for quality control of variants that might occur during storage or between different batches, but also for a critical evaluation of protein drug candidates during early development. The analysis of endogenous protein constructs can improve the understanding, but also the diagnosis of human diseases, as various diseases arise from protein misfolding, aggregation, or modifications, such as diabetes, neurodegenerative diseases, or cancer [7–10].

CZE can be hyphenated to MS using either a sheath liquid (SL) or a sheathless interface. A SL interface offers the advantage that, depending on the selected SL composition, subsequent MS analysis can be performed under either denaturing or native-like ESI conditions. Both approaches provide information about the composition of the proteoforms separated by CZE. Native ESI conditions can maintain the higher-order structure of a protein complex and allow for structural analysis of separated proteoforms. Denaturing ESI conditions typically lead to dissociation of the protein complex and enable the analysis of the complex on subunit level, allowing for better detection of small modifications, or simply provide a better signal intensity if native MS analysis is not required. The upfront separation facilitates the detection of low-abundant species, which are often not detected in direct infusion MS experiments due to overlapping MS signals.

Compared to other native separation techniques, like ion exchange chromatography, size exclusion chromatography, or hydrophobic interaction chromatography, CZE requires less sample, provides better sensitivity, and can be applied for affinity-based separations [11]. However, only a few works have been reported for the characterization of proteins and protein complexes by capillary zone electrophoresis-mass spectrometry (CZE-MS) under native-like conditions [2, 12, 13]. Most of these studies show a separation between different proteins, but only a slight or no separation between the proteoforms of one protein construct.

We recently developed a CZE-MS method that enables CZE separation under native-like conditions using an AmAc-based BGE, a static capillary coating based on hydroxypropyl methylcellulose (HPMC), and a nanoSL interface [14]. The performance of the method was demonstrated by CZE-MS analysis of trastuzumab (isoelectric point (pI) ~ 9.1) and NISTmAb (pI ~ 9.2), enabling a powerful separation of the proteoforms of both antibodies.

In this study, we refine the developed CZE-MS method and demonstrate its flexibility for the analysis of proteins having different pIs by varying the pH value of the BGE depending on the pI of the protein, the desired separation performance, and analysis time. In addition, we investigate whether the separation conditions can preserve non-covalent interactions in protein complexes. Therefore, we analyzed the proteoforms of several mAbs with different pIs as well as the protein complex hemoglobin (Hb) by CZE-MS. The pH of the AmAc-based BGE was adapted depending on the respective protein to obtain high resolution of proteoform separation in a reasonable time scale. The separation of typical charge variants was systematically evaluated. For mAb and hemoglobin analysis, a SL with denaturing properties was applied. In addition, different SLs with non-denaturing properties were tested for hemoglobin analysis to preserve the non-covalent interactions of the complex. Based on these results, we provide general recommendations for optimal conditions for CZE-MS analysis of proteins and proteoforms.

## Materials and methods

### Chemicals and materials

Adalimumab (Hulio®) (charge: 1825216; 50 mg/mL), infliximab (charge: 8RMKA90603; 10 mg/mL), and bevacizumab (charge: B8020H05; 25 mg/mL) were purchased from Evidentic GmbH (Berlin, Germany) and stored at −80 °C until use. Human hemoglobin (Hb), (hydroxypropyl methylcellulose) (HPMC), and hydrofluoric acid (HF, 40%(v/v)) were purchased from Merck (Darmstadt, Germany). Isopropanol (IPA, LC-MS grade), acetic acid (HAc), and ammonium acetate (AmAc, ≥ 99%) were obtained from Carl Roth GmbH & Co. KG (Karlsruhe, Germany). Peptide N-glycosidase F (PNGase F) was obtained from Serva (Heidelberg, Germany). All solutions were prepared using ultrapure water (UPW, SG Ultra Clear UV from Siemens Water Technologies, USA). Bare fused silica capillaries (separation capillary: 50 μm inner diameter (ID), 365 μm outer diameter (OD), and sheath liquid (SL) capillary: 100 μm ID, 240 μm OD) were obtained from Polymicro Technologies (Phoenix, AZ, USA).

### Sample preparation

All mAb samples were diluted with UPW to a final concentration of 5 mg/mL for CZE-MS analysis. Adalimumab was additionally digested with PNGase F prior to CZE-MS analysis to reduce the complexity of the drug product. PNGase F (10^6^ u/mL) was diluted with 25 mM ammonium bicarbonate buffer (pH 7.8) to 10 u/µL. Ten microlitres of the diluted PNGase F solution was added to 10 µL adalimumab (10 µg/mL) and incubated at 37 °C for 22 h (250 rpm) to remove the glycans from the Fc part of the mAb. After twenty-two hours, the PNGase F–digested adalimumab sample was analyzed by CZE-MS. Human Hb was diluted in 50 mM AmAc to a concentration of 5 mg/mL. Aliquots were stored at −80 °C until use. For CZE-MS analysis, an aliquot was diluted with 50 mM AmAc to a final concentration of 1 mg/mL.

### CZE-MS analysis

For CZE-MS analysis, an HP ^3D^CE instrument (Agilent Technologies GmbH, Waldbronn, Germany) was coupled to a timsTOF SCP mass spectrometer (Bruker Daltonics, Bremen, Germany). In the timsTOF SCP, the first multipole was replaced by a tunnel. An etched HPMC-coated capillary (length 50 cm, if not stated otherwise) was used for all experiments to avoid protein adsorption. The capillaries were prepared as described recently [14].

If not stated otherwise, a BGE of 50 mM AmAc (pH ~ 6.8) was applied for the analysis of adalimumab and infliximab and a BGE of 19.98 mL 50 mM AmAc adjusted with 19 µL 2 M HAc to pH 6.0 was applied for infliximab, bevacizumab, and hemoglobin. Infliximab was additionally measured using a BGE of 19.78 mL 50 mM AmAc adjusted with 220 µL 2 M HAc to pH 5.0 All samples were injected hydrodynamically using a pressure of 30 mbar for 6 s. Samples were separated using a separation voltage of 20 kV. Before the first run and between runs, the capillary was flushed with BGE for 5 min.

For CZE-MS coupling, the nanoCEasy interface was applied [15]. All emitters were obtained from BioMedical Instruments. The emitter was positioned 5 mm in front of the MS orifice when using a denaturing SL and 3 mm in front of the MS orifice when using a SL with native properties. The position of the emitter was supervised using a digital microscope (Dino-Lite AM7515MZTL, Almere, Netherlands). For all CE-MS analysis, the timsTOF SCP was operated in the TIMS off mode.

All mAbs and hemoglobin were analysed by CZE-MS under denaturing ESI conditions using a SL based on IPA/H_2_O/HAc (50/45/5) and an emitter with a tip length of 3 mm and an opening of 30 µm at the tip. The *m*/*z* range was set to 1000–8000, the transfer time to 150 µs, and the pre-pulse storage to 40 µs. IsCID was set to 60 eV for declustering and a dry temperature of 180 °C was selected.

Hemoglobin was additionally analysed by CZE-MS using native-like ESI conditions. For native ESI-MS analysis, three SL compositions were compared based on 100% H_2_O, H_2_O/IPA (95/5), and H_2_O/IPA (90/10), each containing 10 mM AmAc. Emitters with a tip length of 4 mm and a tip opening of 15 µm were used. An m/z range of 700–6000, a pre-pulse storage of 35 µs, and a transfer time of 130 µs were selected. IsCID was set to 0 eV and the source dry temperature to 65 °C. Further parameters were listed recently [14].

### Data analysis

CE-MS data were processed and deconvoluted using Compass DataAnalysis 6.1.

## Results and discussion

### CZE-MS analysis of mAbs using an antibody-adapted BGE

**Fig. 1:**
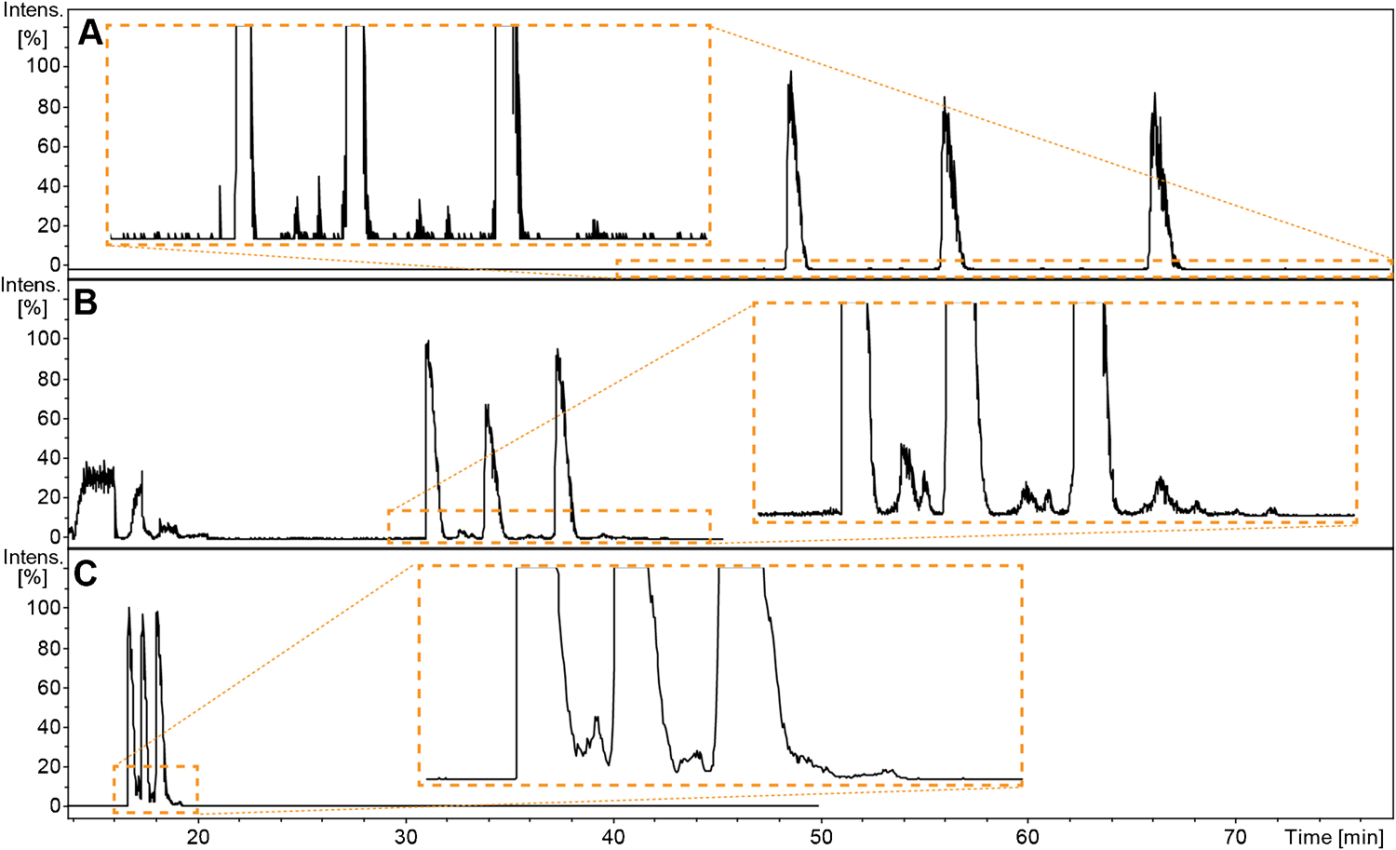
EIEs 3000–4500 of infliximab analyzed by CZE-MS using AmAc-based BGEs with different pH values. **A** BGE: 50 mM AmAc (pH 6.8); **B** BGE: 50 mM AmAc adjusted with HAc to pH 6.0; **C** BGE: 50 mM AmAc adjusted with HAc to pH 5.0; conditions: HPMC-coated capillary (50 cm), 20 kV, SL: IPA/H_2_O/HAc (50/45/5)

We recently presented a CZE-MS method where we applied a neutral capillary coating and a BGE of 50 mM AmAc with a pH of about 6.8, resulting in a high separation performance of the charge variants of trastuzumab (pI ~ 9.1) and NISTmAb (pI ~ 9.2) [14]. However, no pH adjustment was made. Thus, we evaluated the influence of the BGE’s pH value on the separation performance for CZE-MS analysis of different antibodies of a wide range of pI (7.6–9.2). The pH of the BGE was adjusted by the addition of acetic acid to 50 mM AmAc (Fig. S1). In general, a pH value close to the pI reduces the net charge of the mAb and thereby increases the electrophoretic resolution between charge variants. Using a BGE of 50 mM AmAc (pH = 6.8) for CZE-MS analysis of infliximab (pI ~ 7.6) results in a high electrophoretic resolution between the different charge variants (Fig. 1A).

However, analysis time is increased to about 75 min, taking almost twice as long as for CZE-MS analysis of trastuzumab or NISTmAb [14]. When we adjusted the pH of the BGE with HAc to pH 6.0, the analysis time was reduced to about 45 min (Fig. 1B). In addition, minor charge variants are visible between the three major charge variants, which can hardly be recognized for the BGE with pH 6.8. A separation at pH 5.0 reduces the separation time by about half to 20 min and the three major proteoforms are still baseline separated (Fig. 1C). However, the separation performance between minor charge variants decreased compared to pH 6.0. For a detailed characterization of infliximab, the separation at pH 6.0 was the best compromise between time, intensity, and separation performance. As shown for infliximab, an AmAc-based BGE with a pH of 6.8 is not the best choice for every mAb and the pH of the BGE should be adapted depending on the protein being analyzed if a high separation performance in a reasonable time is desired. In general, the HPMC-based coating allows CZE separation for a pH value between 2 and 7. A separation in the basic pH range leads to a degradation of the HPMC coating.

**Fig. 2:**
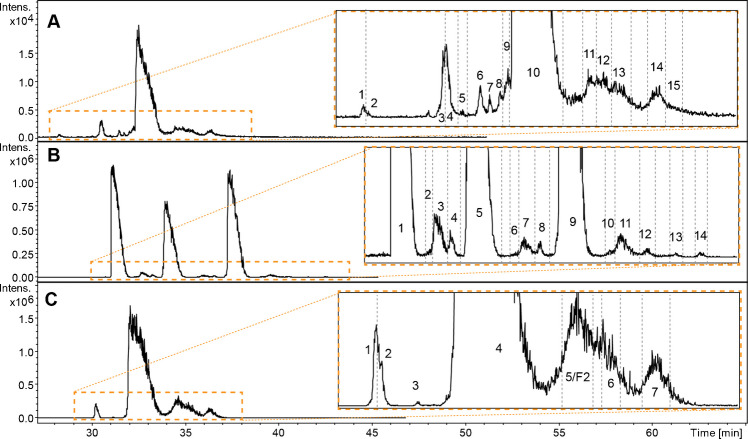
EIEs (3000–4500) of different mAbs analyzed by CZE-MS using AmAc-based BGEs with protein-adapted pH values. **A** Adalimumab (pI: 8.9), BGE: 50 mM AmAc (pH 6.8); **B** infliximab (pI: 7.6), BGE: 50 mM AmAc (pH 6.0); **C** bevacizumab (pI: 8.3), BGE: 50 mM AmAc (pH 6.0). Further conditions: HPMC-coated capillary (50 cm), 20 kV, SL: IPA/H_2_O/HAc (50/45/5)

Apart from infliximab, we analyzed adalimumab and bevacizumab and obtained an optimal separation for adalimumab (pI ~ 8.9) using a BGE of 50 mM AmAc (pH 6.8) and for bevacizumab (pI ~ 8.3) using a BGE of 50 mM AmAc adjusted with HAc to pH 6.0 (Fig. 2). A SL with denaturing properties consisting of IPA/H_2_O/HAc (50/45/5) was applied for all mAbs as it provides higher signal intensities and better spectra quality compared to a SL with native properties.

### Characterization of adalimumab, infliximab, and bevacizumab

*Adalimumab* (Hulio®) shows a high charge heterogeneity with various basic variants (peaks 1–9) and a few broad acidic peaks (peaks 11–15) (Fig. 2). Corresponding mass spectra of peaks 1–15 can be found in the supporting information (Fig. S2) and a list containing all detected proteoforms can be found in Table S1. Basic variants detected for adalimumab can be assigned as species with one (peak 4) and two (peak 2) unprocessed lysine, one (peak 3) and two (peak 1) C-terminal proline amidations, a succinimide residue (peak 6), and an N-terminal aspartic acid loss as well as low-abundant variants with both a C-terminal proline amidation and additionally one N-acetylneuraminic acid (NANA; peak 7). We point out that the variant with two proline amidations (peak 1) and the variant with a loss of aspartic acid (peak 7) have a mass difference of approximately −116 Da and  approximately −115 Da, respectively, relative to the main form, not resolvable by MS on the intact level. However, CZE allows a clear separation between the doubly amidated species carrying two additional positive charges and the species with aspartic acid loss carrying only one additional charge, enabling a distinction of these two species by their migration order. In the acidic region, masses indicate variants carrying one NANA (peak 11) and two NANAs (peak 14), glycated variants (peak 15), as well as variants with a single deamidation. In peak 14, a mass difference of about 1900 Da was observed (Table S1), remaining after PNGase F treatment, which might indicate variants with an additional Fab glycosylation, presumably containing (Fuc)_1_(GlcNAc)_2_(Man)_3_(GlcNAc)_2_(Gal)_1_(NeuGc)_1_ (G1FS). Additionally, we detected unknown variants including basic species with modifications of about +68.9 Da (peak 5), −15.2 Da (peak 8), and −42.3 Da (peak 9) as well as an acidic variant with a mass increase of ~170.3 Da. The modifications of +68.9, Da, −42.3 Da, and +170.3 Da remain after PNGase F treatment, indicating a modification in the primary sequence of the mAb (Fig. S3). In total, about 100 proteoforms can be detected for adalimumab (Hulio®). Several variants, including potential double proline amidation or aspartic acid loss, have already been reported for another adalimumab product, Humira®, using cation exchange chromatography-MS [16]. We additionally detected variants with single proline amidation, Fab glycosylation, and variants with sialic acids. Furthermore, we were able to assign the double amidated species and species with an N-terminal Asp loss more reliably due to a better separation between these variants differing in one charge [16].

*Infliximab* (Remicade®) shows highly abundant lysine variants (Fig. 2 and Fig. S4; peaks 1, 5, 7). After each of the three lysine variants (2K, 1 K, 0 K), acidic species of the respective lysine variants migrate, including species with an additional single deamidation (peaks 4, 8, and 12–14), N-glycolyl neuraminic acids (NGNA) (peaks 2, 6, and 10), as well as variants with a mass difference of approximately + 268 Da to the main glycoforms of each lysine variant (peaks 3, 7, and 11). The change in the ratio between the main glycoforms suggests that the modification of + 268 Da occurs on the N-glycans of the mAb. Previous studies that investigated the glycan profiles of infliximab detected a glycan containing (Fuc)_1_(GlcNAc)_2_(Man)_3_(GlcNAc)_1_(Gal)_1_(NeuGc)_1_ (G1FS-HexNAc) [17, 18]. This suggests that the variants in peaks 3, 7, and 11 contain a combination of, e.g., G1FS-HexNAc and G0F (for mass 149,038.6 Da) or G1FS-HexNAc and G1F (for mass 149,038.6 Da). About 90 proteoforms were detected for infliximab, which can be found in Table S2.

*Bevacizumab* presents a low charge heterogeneity of about 45 proteoforms with three basic peaks which can be attributed to variants with one unprocessed C-terminal lysine (peaks 1–2) and variants with a succinimide residue (peak 3), as well as a broad acidic peak indicating a single deamidation (peaks 5–7) (Fig. 2, Fig. S5, and Table S3). It is striking that the intensities of the lysine glycoforms in peak 1 change in peak 2, indicating an overlay with another glycoform distribution.

Overall, we demonstrate that the developed CZE-MS method allows the detection of various proteoforms for different antibodies with different pIs and heterogeneity. The method allows a baseline separation between different lysine variants (2K, 1 K, 0 K) for all mAbs analyzed so far. Regarding acidic variants, species containing one or two sialic acids, as well as species with one, two, or three deamidations are baseline separated from each other. Masses that suggest the presence of succinimide variants migrate baseline separated between the 1 K variant and the main form. Variants containing one or two unprocessed lysine and additionally a sialic acid or a deamidation are detected between different lysine variants. Furthermore, the high resolution in CZE allows the distinction between almost isobaric variants differing in the charge (2× proline amidation and aspartic acid loss), which cannot be differentiated by MS on the intact level without separation. The high separation performance in combination with a sensitive ESI-MS analysis enables the detection of about 100 proteoforms for adalimumab, 90 for infliximab, and 45 for bevacizumab (see Tables S1, S2, and S3), as well as 115 for NISTmAb and 70 for trastuzumab, as reported recently [14]. To the best of our knowledge, several charge variants detected for these mAbs with the presented CZE-MS method on the intact level have not been reported before [16, 19–21]. Table 1 shows an overview of all antibody charge variants detected with the developed CZE-MS method (NISTmAb, trastuzumab, adalimumab, infliximab, and bevacizumab) [14].

**Table 1:** Overview of detected charge variants for NISTmAb, trastuzumab, adalimumab, infliximab, and bevacizumab. Glycoforms detected for each charge variant are not listed. All listed variants are suggested based on the intact mass detected in CZE-MS analysis

Suggested charge variant	NISTmAb	Trastuzumab	Adalimumab	Infliximab	Bevacizumab
2 lysine	x	-	x	x	-
1 lysine	x	x	x	x	x
2 lysine, 1× deamidation	-	-	-	x	-
1 lysine, 1× deamidation	x	-	-	x	-
2 lysine + sialic acid	-	-	-	x	-
1 lysine + sialic acid	x	-	-	x	-
2× pro amidation	-	-	x	-	-
1× pro amidation	-	-	x	-	-
1× pro amidation + sialic acid	-	-	x	-	-
N-terminal Asp loss	-	-	x	-	-
N-terminal Gln	x	-	-	-	-
Succinimide	-	x	x	-	x
One sialic acid	x	x	x	x	-
Two sialic acids	-	x	x	-	-
Single deamidation	x	x	x	x	x
Double deamidation	-	x	-	-	-
Triple deamidation	-	x	-	-	-
Fab glycosylation	x	-	x	-	-
Glycation	-	-	x	-	-

**Fig. 3:**
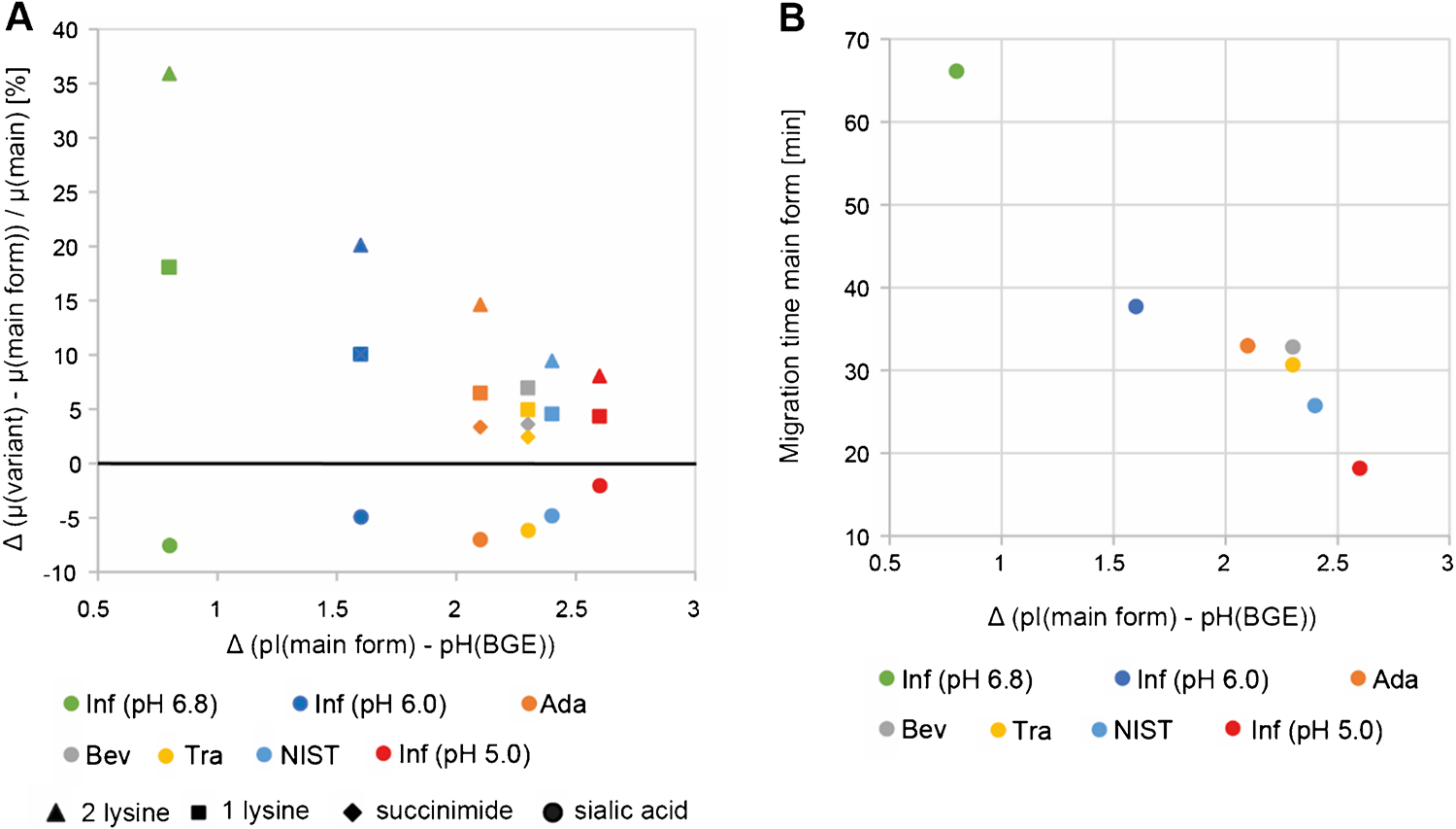
**A** Relation between the mobility of typical variants relative to the main form and the difference between the pI of the mAb and the pH value of the BGE. **B** Relation between the migration time of the main form and the difference between the pI of the mAb and the pH value of the BGE. Abbreviations: Inf, infliximab; Ada, adalimumab; Bev, bevacizumab; Tra, trastuzumab; NIST, NISTmAb

In order to study the achievable resolution systematically, we evaluated the mobility differences for frequently observed charge variants. In Fig. 3A, the relative mobility differences of frequently observed variants are plotted against the difference between the pI of the mAb and the pH of the BGE.

Clearly, the resolution increases for the lysine variants with a smaller difference between the pI of the mAb and the pH of the BGE. This behavior corresponds to the expected charge differences of the mAb at the different pH values of the BGE. Indeed, when assuming a charge difference of + 1 for each additional lysine and the mobility dependence on charge to be the same as under acidic conditions (see Eq. 1) [22], the charge can be calculated for each pair of lysine variants. In this way, a charge of 9.6 was calculated for the main form of NISTmAb and a charge of 6.1 for the main form of adalimumab (at pH_BGE_ 6.8). In this charge range, even small differences in charge (< 1) are sufficient to achieve baseline separation between proteoforms.

1 $$\mu_{ef}=\frac{x\times In\left(1+0.35Q\right)}{M^{0.411}}$$

Interestingly, the often-observed succinimide variant shows a mobility shift of only 30–50% of the one observed for the lysine variants of the same mAb (diamonds in Fig. 3). Sialic acids may behave differently depending on the respective mAb. For instance, for infliximab, a smaller mobility shift compared to the lysine variant is observed.

In general, we recommend choosing a BGE with a pH value of 2.0–2.5 below the pI of the mAb, depending on the heterogeneity of the mAb, to obtain a high separation performance. This typically results in an analysis time of less than 40 min (Fig. 3B). A pH value that is closer than 1.5 to the protein’s pI value results in longer analysis times, and additional information that can be obtained due to a higher electrophoretic resolution is limited. For some applications, a baseline separation between the different lysine variants might be enough. Here, a more acidic BGE can be used, which results in a higher net charge of the protein, allowing for a faster CZE-MS analysis of the drug product as described above for CZE-MS analysis of infliximab at pH 5.0.

### Native CZE-MS analysis of the protein complex hemoglobin

**Fig. 4:**
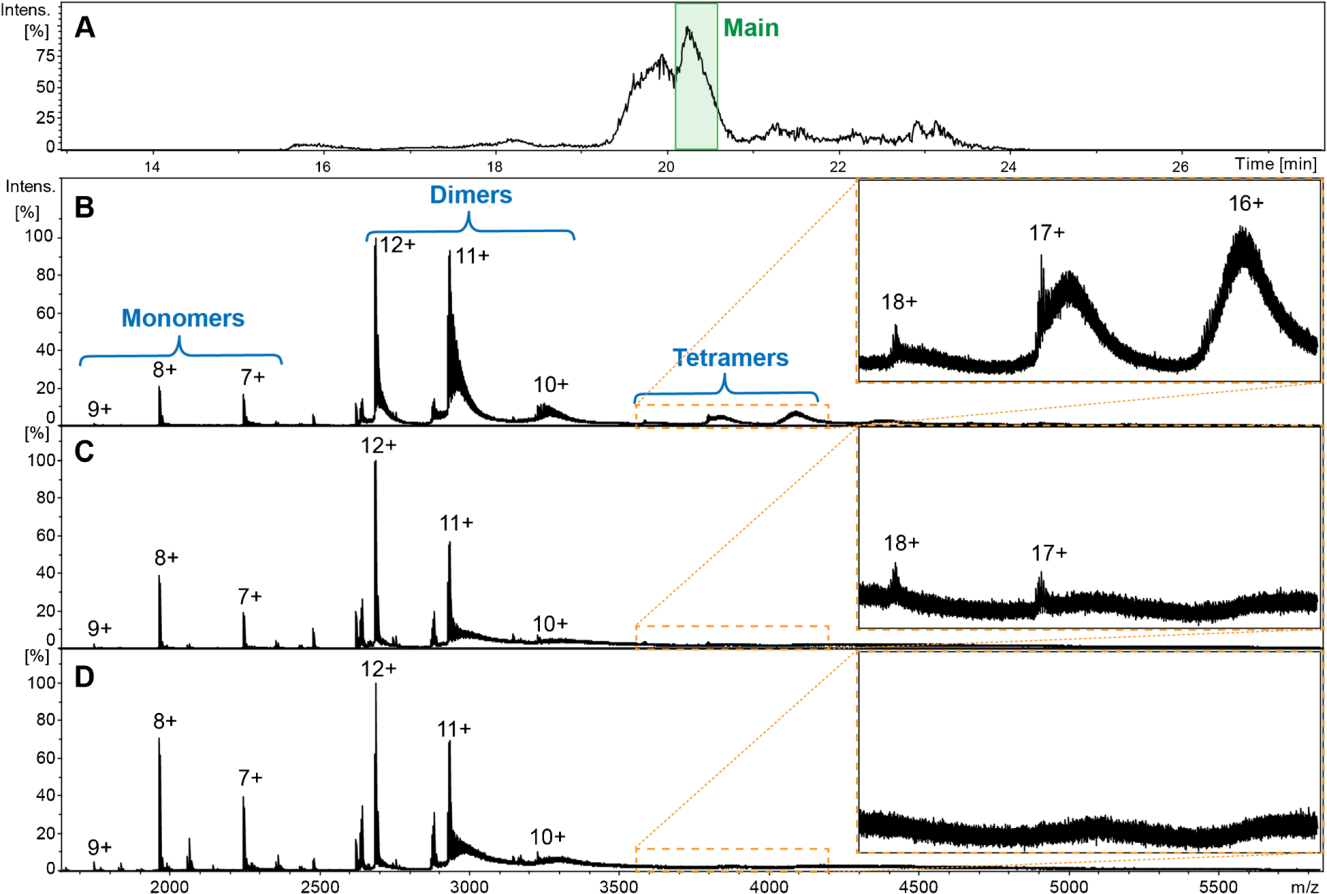
EIE and mass spectra of the main form of Hb analyzed by CZE-MS using different SL compositions. **A** EIE 1900–3000; **B** SL: 100% H_2_O, 10 mM AmAc; **C** SL: H_2_O/IPA (95/5), 10 mM AmAc; **D** SL: H_2_O/IPA (90/10), 10 mM AmAc. Conditions: HPMC-coated capillary (50 cm), BGE: 50 mM AmAc (pH 6.0), 20 kV

Beyond the analysis of proteoforms of monomeric proteins, native CE-MS can provide valuable information regarding the structure and stoichiometry of protein complexes [3–5, 12]. To investigate the performance of the presented CZE-MS method for analyzing protein complexes, we analyzed human hemoglobin (Hb) as a model protein complex. Hb is a non-covalently assembled tetramer composed of two α-chains (MW(α): 15126.38) and two β-chains (MW(β): 15867.24). Each subunit is non-covalently bound to a heme b-group (MW: 616.5 Da), resulting in an average molecular weight of 64453.24 Da for the tetramer (α_2_β_2_h_4_). For CZE separation of Hb (pI: ~ 7), we used a BGE of 50 mM AmAc adapted with HAc to pH 6.0. For coupling to MS, a denaturing SL (IPA/H_2_O/HAc (50/45/5)) as well as a SL with native properties was applied to preserve the non-covalent interactions of the tetramer. To obtain native-like ESI conditions, we tested three SL compositions based on 100% H_2_O, H_2_O/IPA (95/5), and H_2_O/IPA (90/10), each containing 10 mM AmAc. Since emitters with an emitter tip of 30 µm did not produce a stable spray at less than 10% IPA in the SL, we applied emitters with an emitter tip of only 15 µm for all three SL compositions. CZE-MS analysis of Hb results in high-abundant charge states of dimers for each of the three SL (Fig. 4).

The abundance of the monomers increases with increasing percentage of IPA in the SL. Charge states of the tetramer are detected with low abundance for the SLs containing no IPA and 5% IPA, but completely disappear for the SL containing 10% IPA. Typically, more dimer and monomer ions than tetramer ions are observed for commercial Hb compared to fresh human Hb [23, 24]. Since direct infusion of Hb (0.1 mg/mL in 100 mM AmAc) also resulted in high intensities of the charge states of monomers, dimers, and tetramers (Fig. S6), we assume that the low abundance of the tetramer is not only due to dissociation of the complex during CZE-MS analysis but also due to already high levels of dimers and monomers in the sample. When comparing the mass spectra of Hb obtained by direct infusion and by CZE-MS, it is noticeable that the charge states are better declustered for the data obtained by CZE-MS. CZE provides the advantage that unbound salts are separated from the protein, resulting in better declustering and therefore, a more accurate mass measurement of the analyte.

### Characterization of Hb variants

**Fig. 5:**
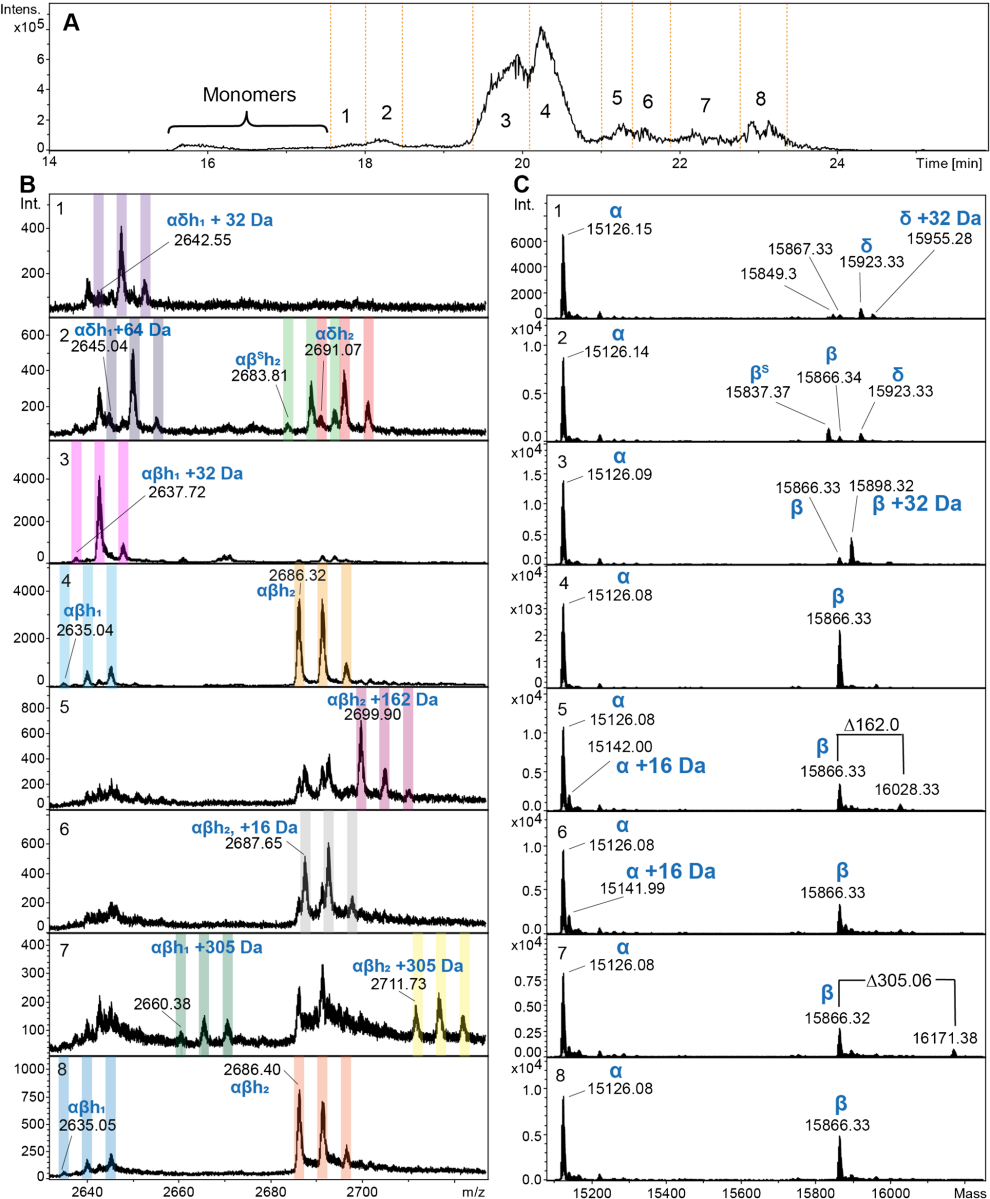
CZE-MS analysis of Hb using a SL with native and denaturing properties. **A** EIE 1900–3000 and **B** mass spectra of charge state 12+ using a native SL based on H_2_O/IPA (95/5), 10 mM AmAc, and a HPMC-coated capillary (50 cm); adduct peaks are marked with the same colour. **C** Corresponding deconvoluted mass spectra using a denaturing SL based on IPA/H_2_O/HAc (50/45/5) and a HPMC-coated capillary (60 cm). CE conditions: BGE: 50 mM AmAc (pH 6.0), 20 kV

We characterized Hb based on CZE-MS measurements using the SL containing 95% H_2_O, 5% IPA, and 10 mM AmAc, as this SL composition provides a better spectra quality with fewer adduct ion peaks than the SL containing no IPA. Mass spectra dimers of separated variants are shown in Fig. S7 and the zoom-in mass spectrum of the 12+ charge state in Fig. 5B. In general, we observed adduct peaks of + 62.0 Da with varying intensity for all variants when using a native-like SL in CZE-MS analysis of Hb that were not observed in direct infusion experiments of Hb under native-like conditions. We assume that these adduct peaks are formed during CZE separation and the mass of 62.0 Da could be attributed to a H_2_CO_3_ adduct. Assignment of Hb variants was additionally verified by CZE-MS analysis using a denaturing SL (IPA/H_2_O/HAc (50/45/5)) (Fig. 5C). The denaturing SL leads to dissociation of Hb dimers into their subunits, enabling a more reliable assignment of modifications and their localisation to the alpha or beta chain.

CZE-MS analysis of Hb revealed 8 peaks being separated in CE, each showing different proteoforms of Hb, with the dimer containing two heme (αβh_2_) being the most abundant proteoform (peak 4) (Fig. 4A). In the following section, detected Hb variants are discussed for the native-like SL. The denaturing SL serves as support for the assignment of variants.

Isotopic patterns in peak 4 can be assigned as dimers with two heme each containing Fe^3+^. A heme containing Fe^3+^ carries one positive charge. Consequently, charge states of the dimer αβh_2_, for instance, charge state 12+ appear as [M+10H]^12+^ instead of [M+12H]^12+^, as demonstrated in Fig. S8. Deconvolution of the native mass spectra results in wrong masses as the deconvolution algorithm adds the number of protons according to the number of charges, thus, not taking charges from oxidized iron in the heme into account. Therefore, raw mass spectra of Hb variants of charge state 12+ are shown for the native-like SL (Fig. 5B). Corresponding manually deconvoluted masses are listed in Table 2.

**Table 2:** Manually deconvoluted masses detected in peaks 1–8 (Fig. 5) in CZE-MS analysis of Hb and assigned variants. Manual deconvolution was performed based on charge state 12+, assuming that in peaks 1–7 all heme contain Fe^3+^ and in peak 8 the αβh_2_ dimer contains one Fe^2+^ and one Fe^3+^

Peak	m/z of charge state 12+	Measured deconvoluted mass	Assigned variant	Theoretical mass of assigned variant
1	2642.55	31,700.6	αδh_1_+32 Da	31,698.8
2	2645.04	31,730.48	αδh_1_+64 Da	31,730.8
	2683.81	32,195.72	αβ^s^h_2_	32,196.23
	2691.07	32,282.84	αδh_2_	32,283.29
3	2637.72	31,642.64	αβh_1_+32 Da	32,641.72
4	2635.04	31,610.48	αβh_1_	31,609.72
	2686.32	32,225.8	αβh_2_	32,226.21
5	2699.90	32,388.8	αβh_2_+162 Da	32,388.21
6	2687.65	32,241.8	αβh_2_+16 Da	32,242.21
7	2660.38	31,914.56	αβh_1_+305 Da	31,915.02
	2711.73	32,530.76	αβh_2_+305 Da	32,531.51
8	2635.05	31,609.6	αβh_1_	31,609.72
	2686.40	32,225.8	αβh_2_	32,226.21

The broad signals migrating before peak 1 mainly contain the α-subunit, enhancing the assumption that monomers are already present in the sample. Low-abundant signals in peaks 1 and 2 indicate dimers containing the delta-chain (αδh_2_). Hb D is a minor Hb variant, which occurs at ~ 3% in adults. The migration time of peak 2, as well as the detected molecular weight of 15,837.37 Da when using the denaturing SL, agrees well with the calculated molecular weight of 15,837.06 for the sickle mutation (β^S^). Low-abundant masses indicating the dimer αβ^S^h_2_ were detected when using the native-like SL. The masses in peak 3 are likely to correspond to an αβh_1_-dimer + 32 Da, which has been reported earlier for commercial human Hb and oxidized fresh human Hb [23]. The denaturing SL allowed localization of this 32 Da modification on the β-chain. The β-chain in human Hb contains two methionine and one cysteine residues, which are easily oxidized [25, 26]. Oxidation of Hb typically leads to a loss of heme [23].

Acidic peaks indicate αβh_2_-dimers with a glycation (peak 5), oxidation (peak 6), and glutathionylation (peak 7), which are almost baseline separated from each other. In peak 8, the isotopic pattern of the αβh_2_-dimers indicates dimers containing one Fe^3+^ and one Fe^2+^, as demonstrated in Fig. S9. The longer migration time of this dimer having one positive charge less than the dimer containing two Fe^3+^ supports this assumption. The dimer in peak 8 could also be attributed to a deamidated variant. However, this can be excluded as deamidation was not detected for the α- and β-subunits when using the denaturing SL.

Overall, the data show that native CZE-MS can preserve non-covalent interactions of Hb when using a SL with 5% IPA or less and allows separation between different Hb variants, such as Hb D, Hb A, as well as glycated, glutathionylated, and oxidized variants. Furthermore, variants that contain iron in different oxidation states (Fe^2+^ and Fe^3+^) can be separated in CE, making the method attractive for a qualitative and quantitative evaluation of the oxidation state of metal ions in protein complexes. Modifications such as glycation, glutathionylation, or oxidation are important biomarkers for diagnosis of diabetes or oxidative stress [27, 28]. Compared to a recently reported denaturing CZE-MS method for Hb analysis [29], native CZE-MS allows the analysis of the overall proteoform population of Hb, including the iron-containing prosthetic heme group. The flexibility of the method to analyze the protein complex on the monomer level using a denaturing SL allows for fast and more precise assignment of proteoforms.

## Conclusion

In our work, we demonstrate the flexibility of the CZE-MS method for the analysis of proteins and protein complexes under native-like separation conditions, as well as both denaturing and native ESI conditions. The ability to adjust the pH value of the BGE between 2 and 7, depending on the protein, the required separation performance, and the desired analysis time, makes the method applicable to a variety of proteins and applications. The charge-based separation allows separation of various proteoforms, thereby enabling the detection of even low-abundant proteoforms as well as the assignment of modifications differing in charge, but not resolvable by MS on the intact level. With respect to mAb characterization, the developed CZE-MS method allows for detailed characterization of different mAbs, as demonstrated for NISTmAb, trastuzumab, adalimumab, infliximab, and bevacizumab, and enables the detection of variants with multiple modifications and proteoforms that have not been reported on the intact level before. Based on the separation of typical charge variants, we recommend choosing a BGE with a pH value of 2.0–2.5 below the pI of a mAb for best charge variant characterization. Applying the AmAc-based separation system, we are able to separate variants of protein complexes as demonstrated here for Hb. When using emitters with a small emitter tip of 15 µm, an AmAc-based SL containing up to 100% H_2_O can be applied. The Hb complex was detected by CZE-MS using a SL containing H_2_O/IPA (95/5), 10 mM AmAc. Variants that contain iron in different oxidation states (Fe^2+^ and Fe^3+^) can be separated in CZE, making the method attractive for a qualitative and quantitative evaluation of the oxidation state of metal ions in protein complexes, which is often interfered by the ESI process. The flexibility to choose between a SL with native or denaturing properties allows for analysis of the whole protein complex as well as for analysis on subunit level, thereby enabling a more reliable assignment of modifications.

Based on this study, we expect the developed CZE-MS method to become a powerful tool for the development and control of therapeutic proteins as well as for studying the proteoforms of protein complexes.

## Supplementary Information

Below is the link to the electronic supplementary material.

Supplementary file1 (PDF 2.57 MB)

## Data Availability

All CZE-MS data are available in the MassIVE repository at https://massive.ucsd.edu/ProteoSAFe/dataset.jsp?accession=MSV000101247.
