# Supplementary material for: Native CZE-MS analysis of antibodies and hemoglobin using protein-adapted CZE and ESI conditions for detailed proteoform characterization
Source: Anal Bioanal Chem. 2026 Jun 13;418(17):5375–86. doi: 10.1007/s00216-026-06611-1 (PMC13473077; doi:10.1007/s00216-026-06611-1)
Supplement: Supplementary file 1 [file 216_2026_6611_MOESM1_ESM.pdf]

Supplementary information:

Native CZE-MS analysis of antibodies and hemoglobin using protein-adapted CZE and ESI conditions for detailed proteoform characterization

Analytical and Bioanalytical Chemistry

*Ann-Katrin Schwenzer, Jule Weiß, Sabrina Buntz and Christian Neusüß**

Faculty of Chemistry, Aalen University, Beethovenstraße 1, 73430 Aalen, Germany

*Corresponding author. E-mail: Christian.neusuess@hs-aalen.de

Table of contents

Supplementary Figures

Figure S1	S-3
Figure S2	S-4
Figure S3	S-5
Figure S4	S-6
Figure S5	S-7
Figure S6	S-8
Figure S7	S-9
Figure S8	S-10
Figure S9	S-11

Supplementary Tables

Table S1	S-12
Table S2	S-17
Table S3	S-21

Supplementary Figures

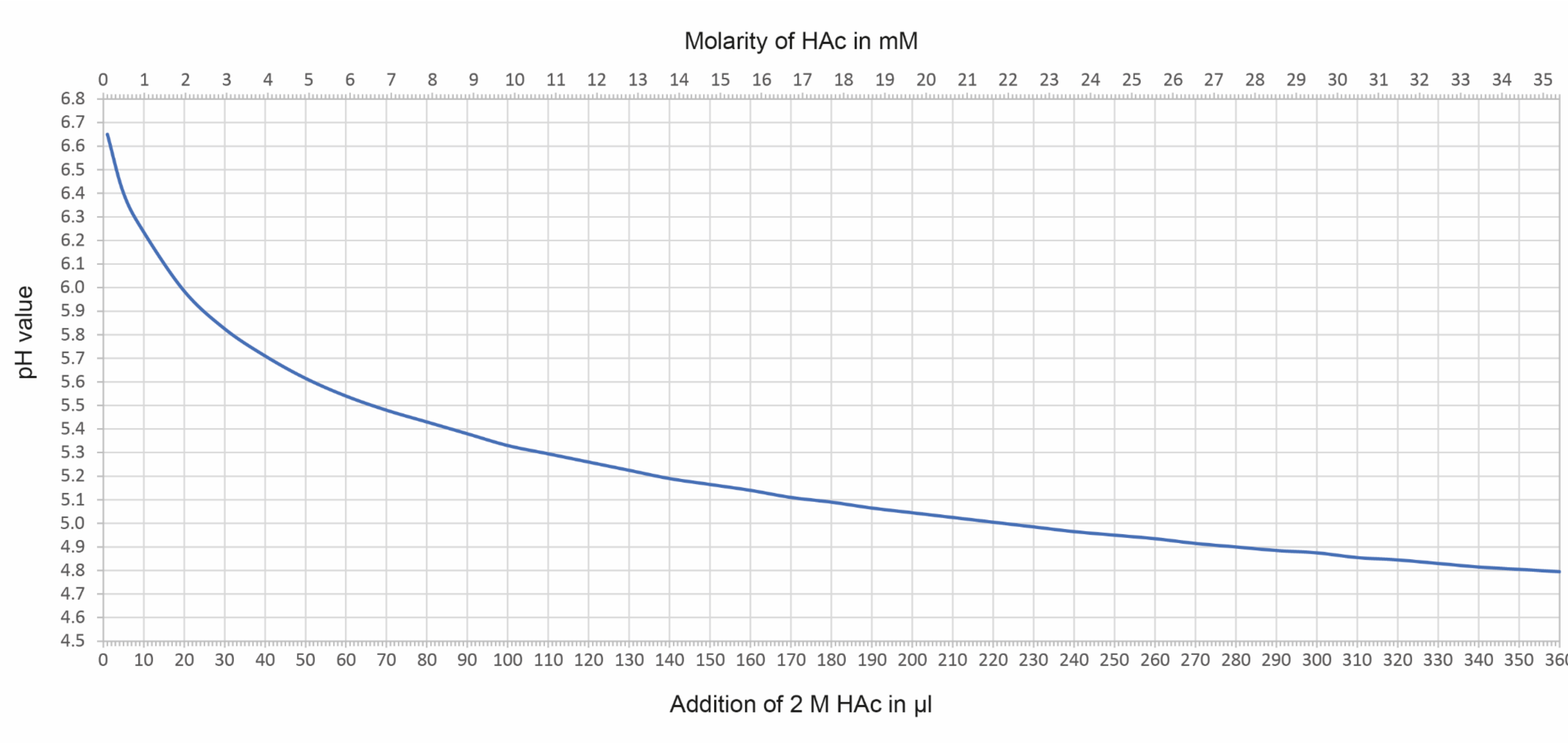

Fig. S1 pH-values obtained when adding 2 M HAc to 20 ml 50 mM AmAc

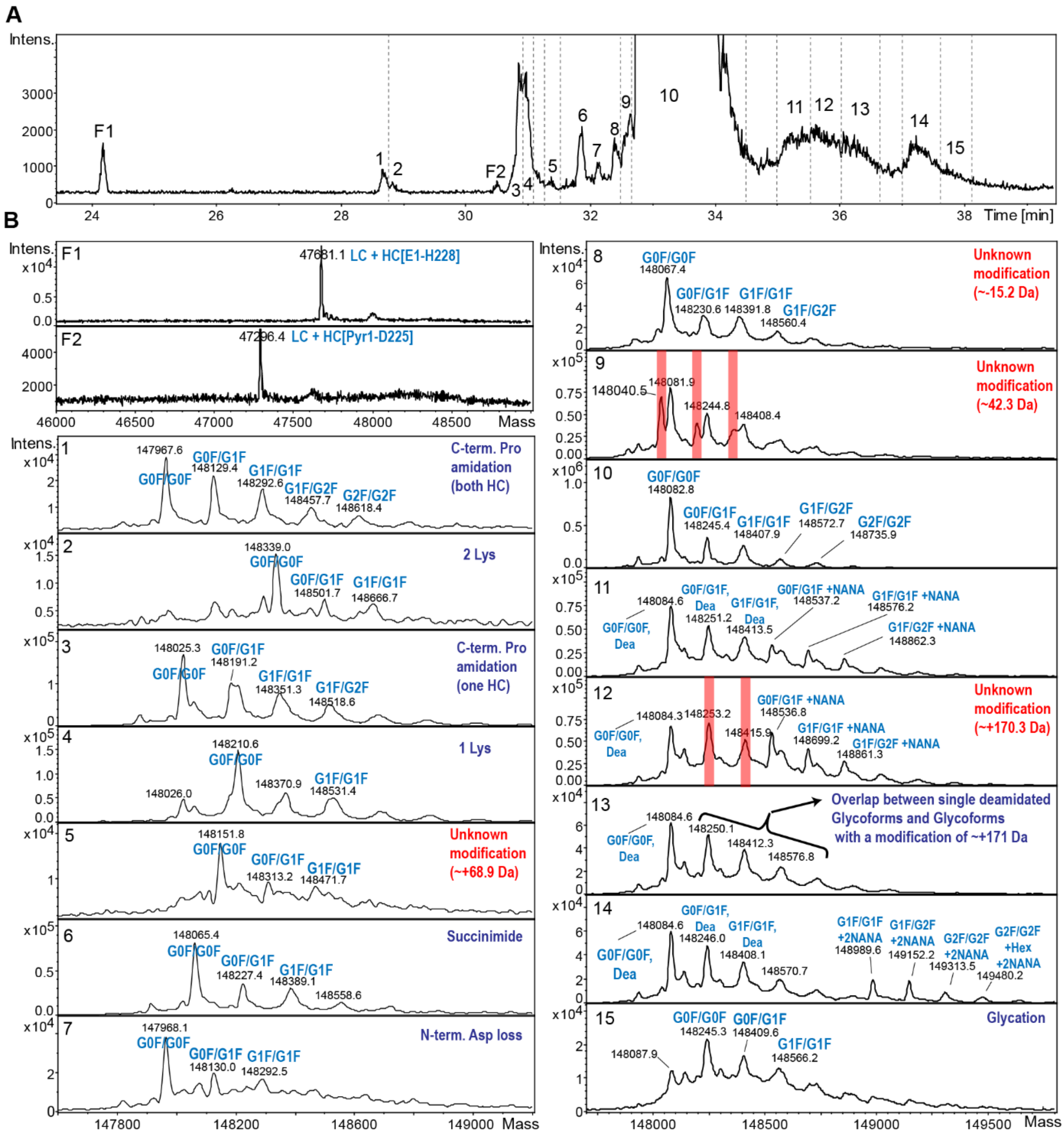

Fig. S2 A) BPE 3000-4500 and B) deconvoluted mass spectra of charge variants obtained from CZE-MS analysis of adalimumab

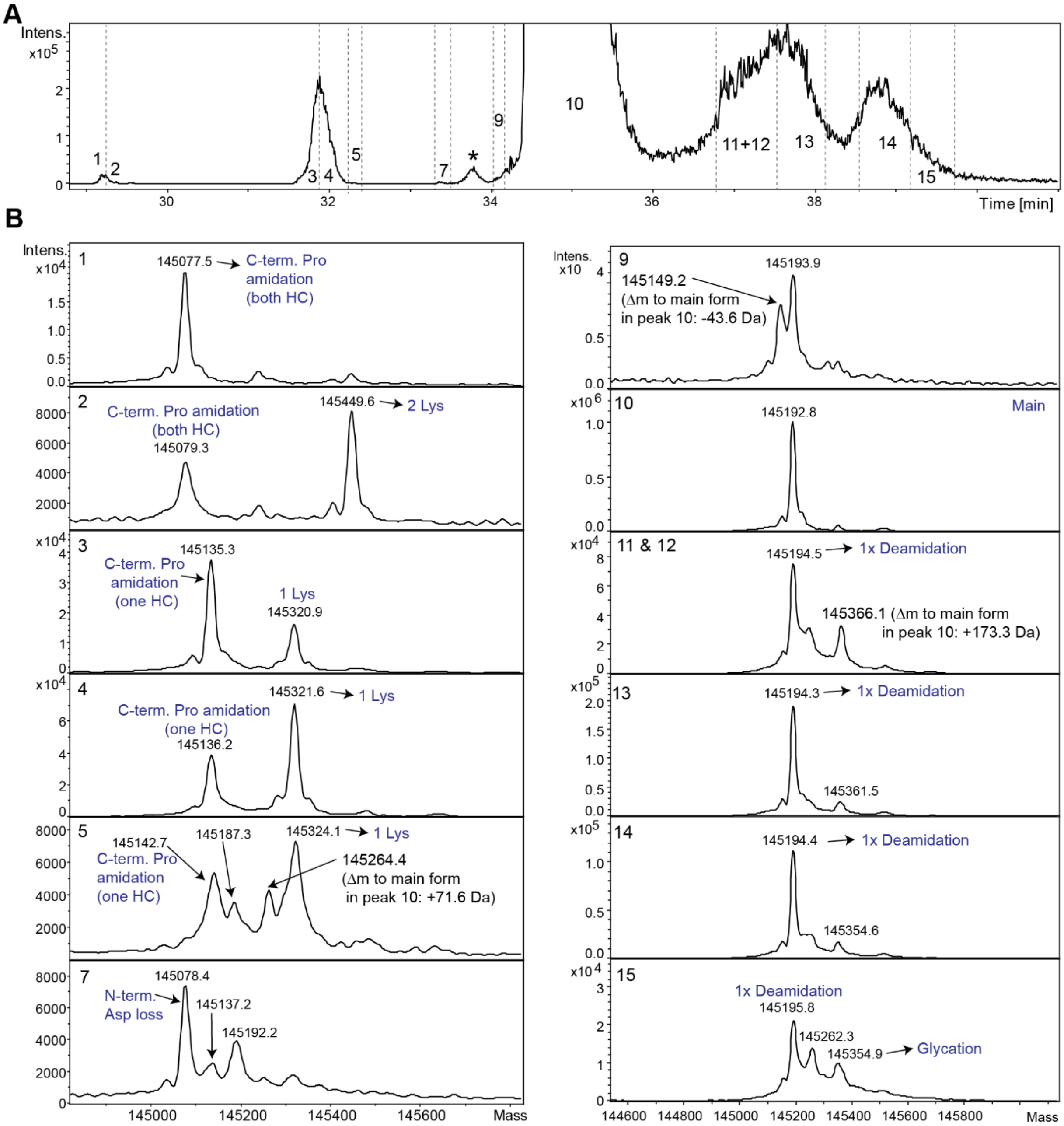

Fig. S3 A) BPE 3000-4500 and B) deconvoluted mass spectra of charge variants obtained from CZE-MS analysis of PNGase F digested adalimumab. The Peak labeled with * in (A) corresponds to monoglycosylated mAb variants, indicating incomplete digest

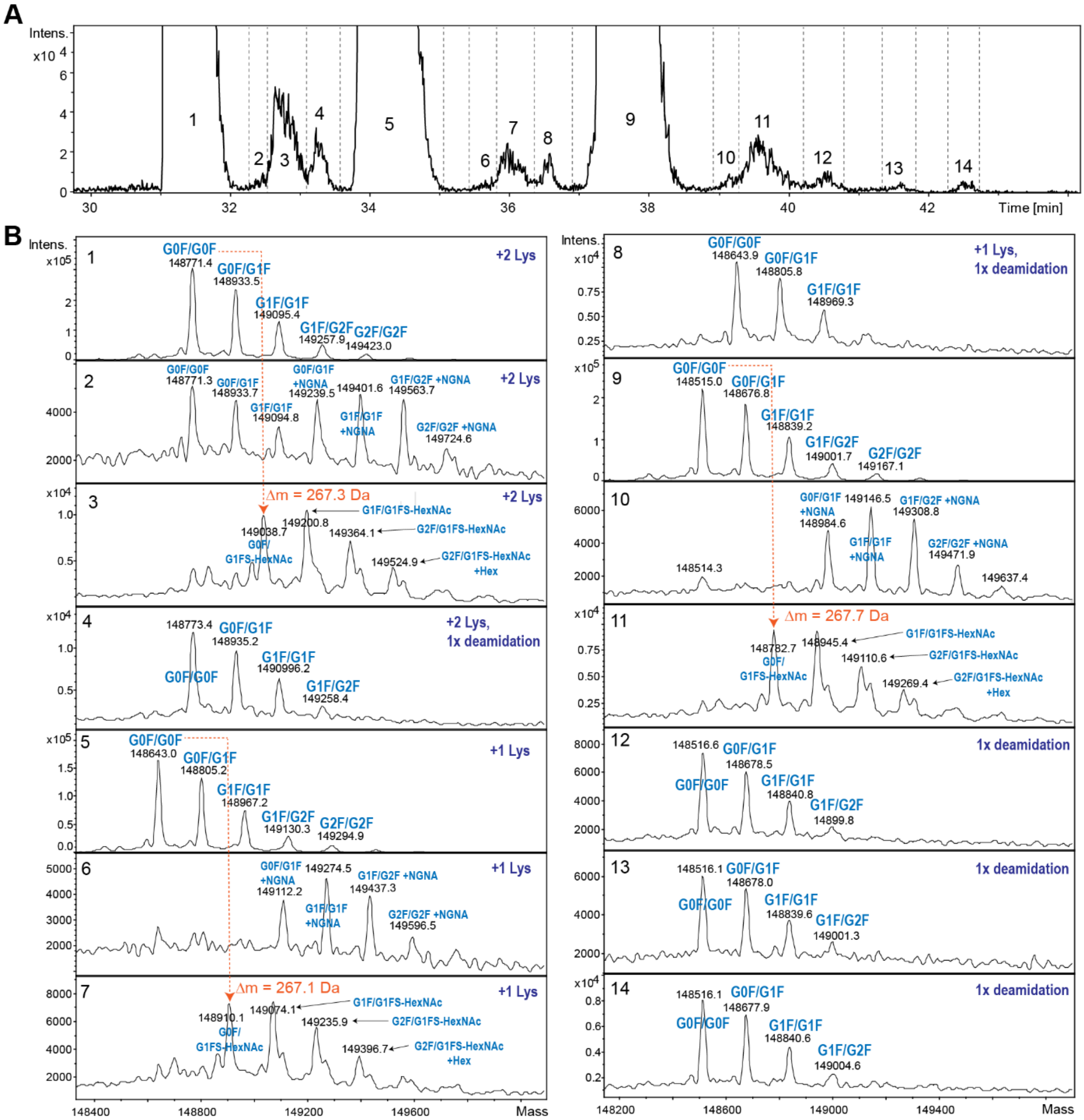

Fig. S4 A) BPE 3000-4500 and B) deconvoluted mass spectra of charge variants obtained from CZE-MS analysis of infliximab. Conditions: HPMC coated capillary (50 cm), BGE: 50 mM AmAc adjusted with 19 µl 2 M HAc to pH 6.0, 20 kV, SL: IPA/H2O/HAc (50/45/5)

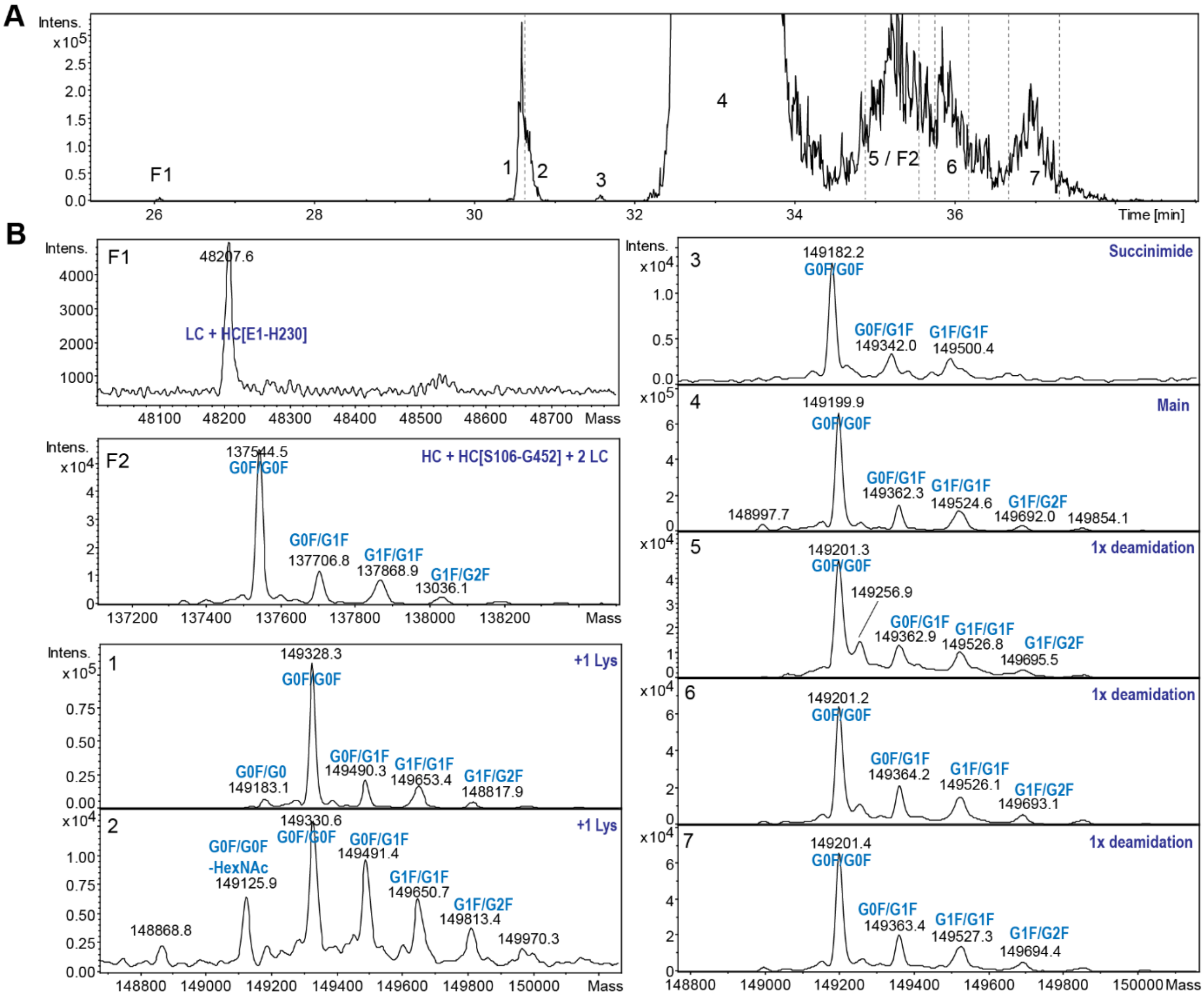

Fig. S5 A) EIC 3000-4500 and B) deconvoluted mass spectra of charge variants obtained from CZE-MS analysis of bevacizumab. Conditions: HPMC coated capillary (50 cm), BGE: 50 mM AmAc adjusted with 19 µl 2 M HAc to pH 6.0, 20 kV, SL: IPA/H2O/HAc (50/45/5)

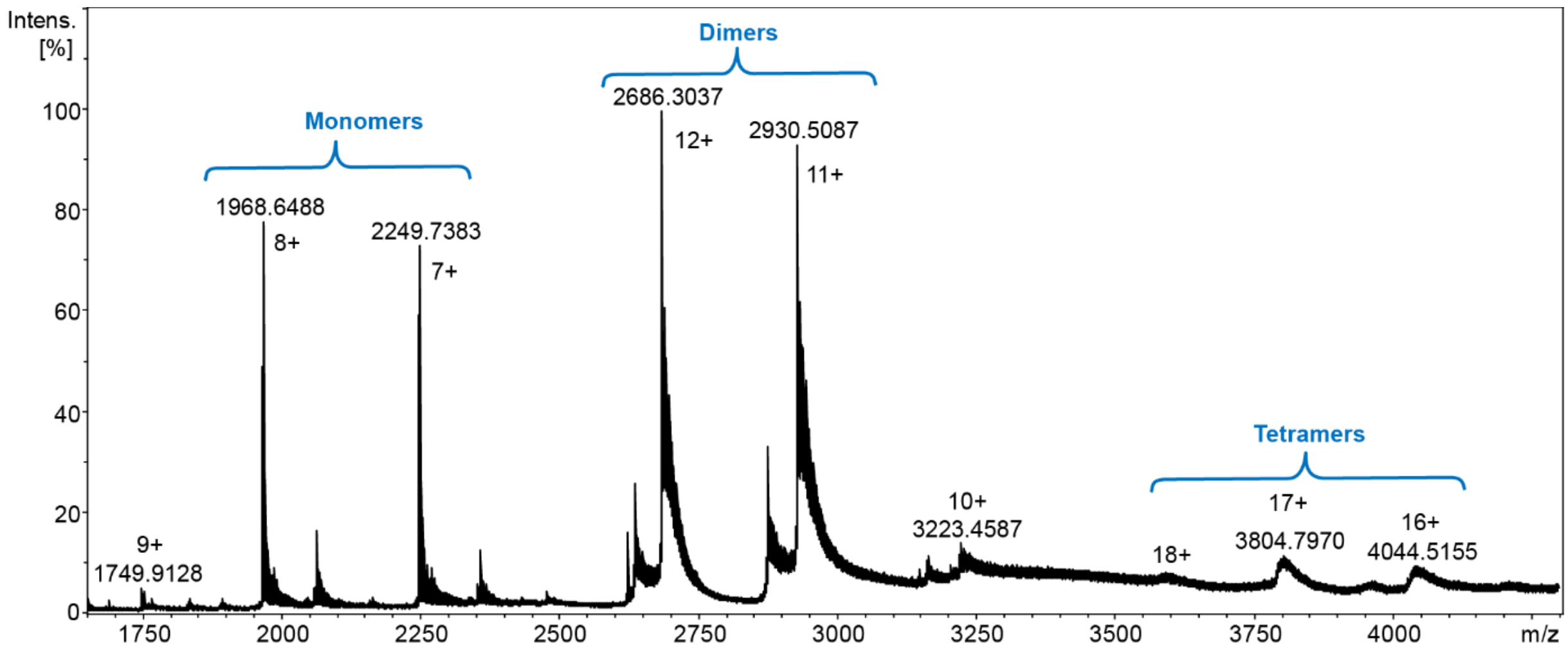

Fig. S6 Mass spectrum of hemoglobin (0.1 mg/ml in 100 mM AmAc (pH~6.8)) analyzed by MS (direct infusion) using the nano ESI source "NEOS" from Bruker

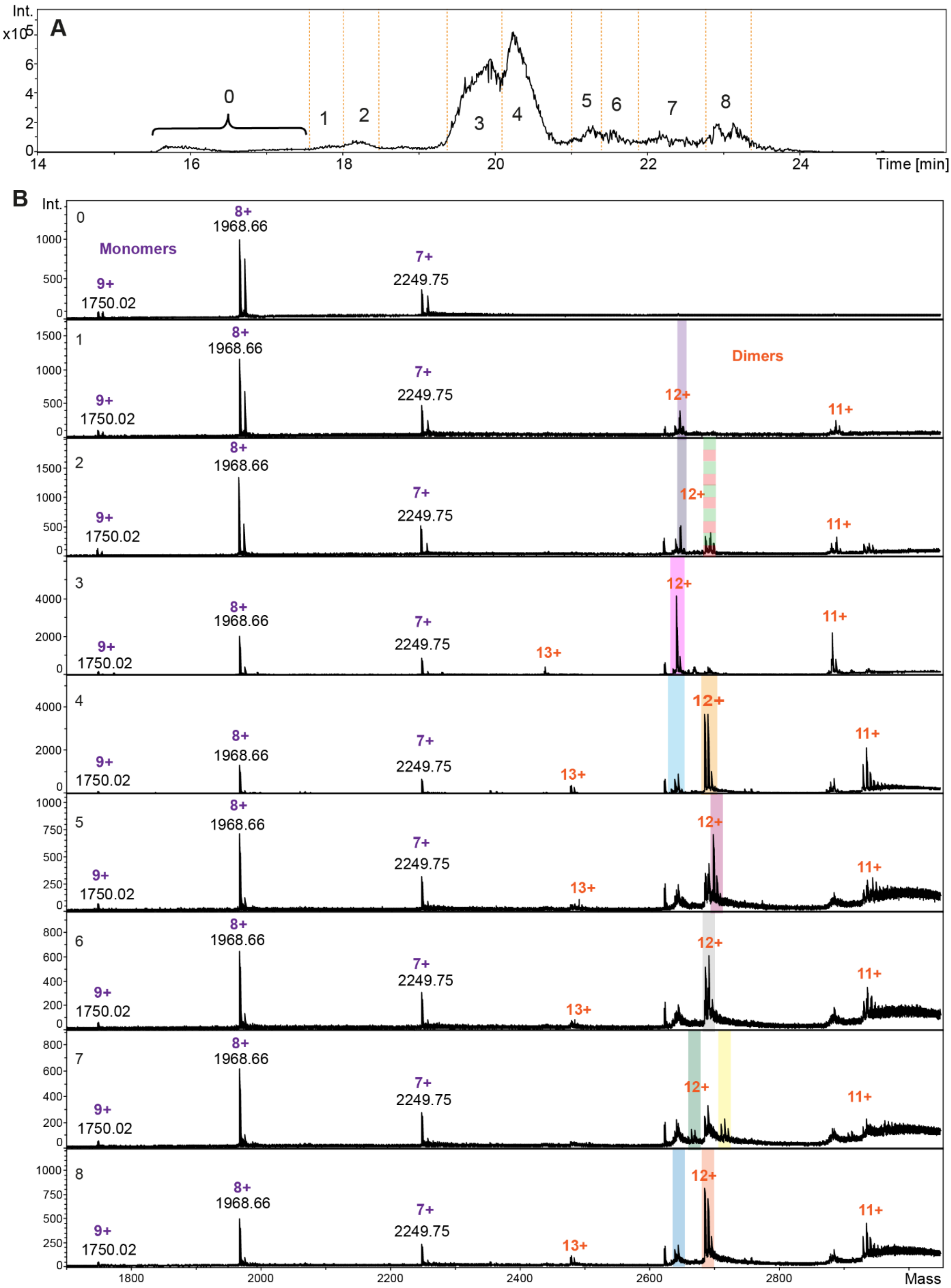

Fig. S7 A) EIC 3000-4500 and B) mass spectra of charge variants obtained from CZE-MS analysis of Hb. Peaks of charge state 12+ are marked with the same color as in Figure 4B. Conditions: HPMC coated capillary (50 cm), BGE: 50 mM AmAc adjusted with 19 µl 2 M HAc to pH 6.0, 20 kV, SL: H_2O/IPA (95/5), 10 mM AmAc

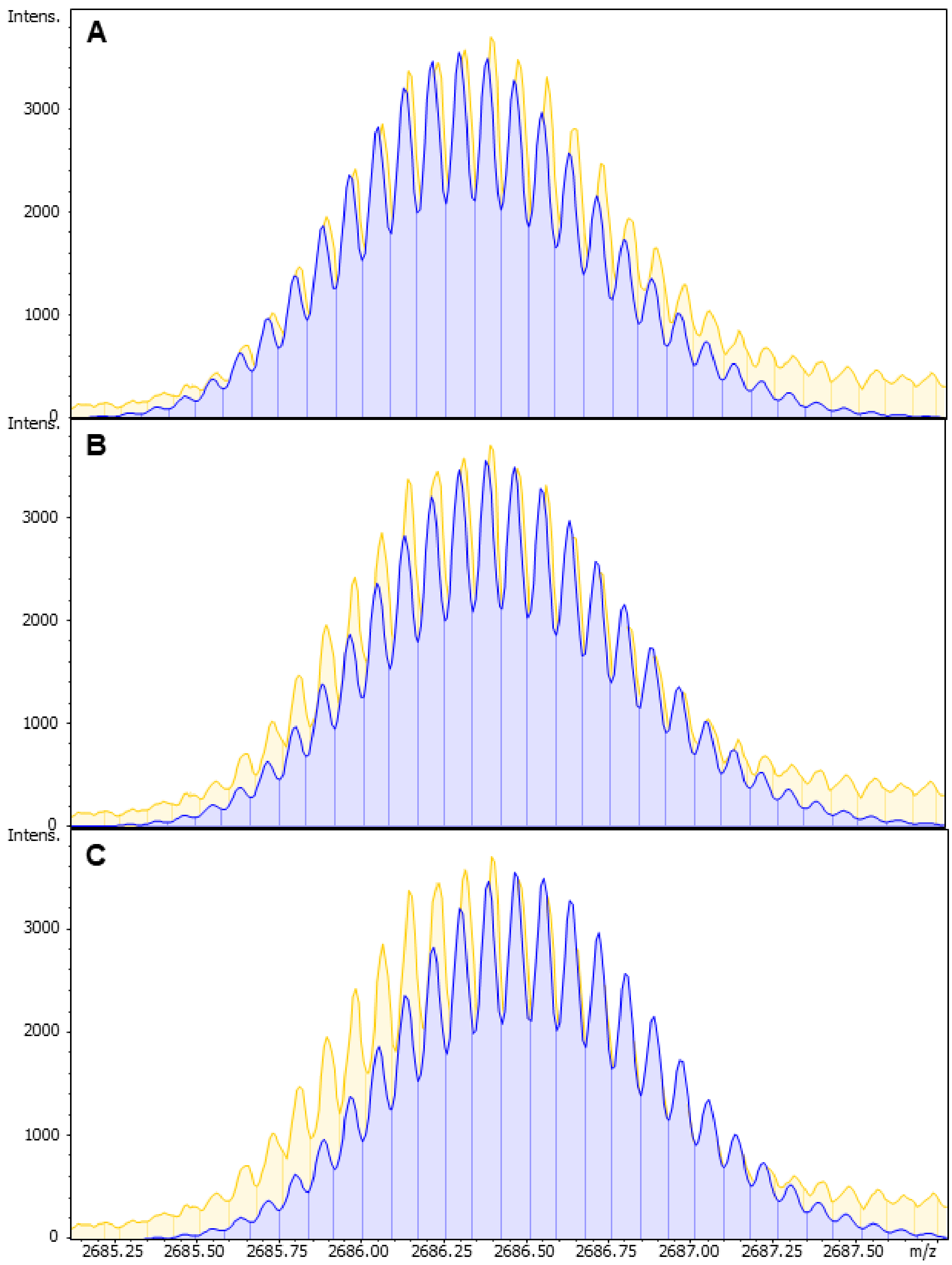

Fig. S8 Overlay of measured (yellow) isotopic pattern of the charge state 12+ of the $\alpha\beta h_2$-dimer in peak 4 with different simulated isotopic patterns of the 12+ charge state of $\alpha\beta h_2$-dimer (M) in blue: (A) $[M+\mathbf{10H}]^{12+}$; (B) $[M+\mathbf{11H}]^{12+}$; (C) $[M+\mathbf{12H}]^{12+}$

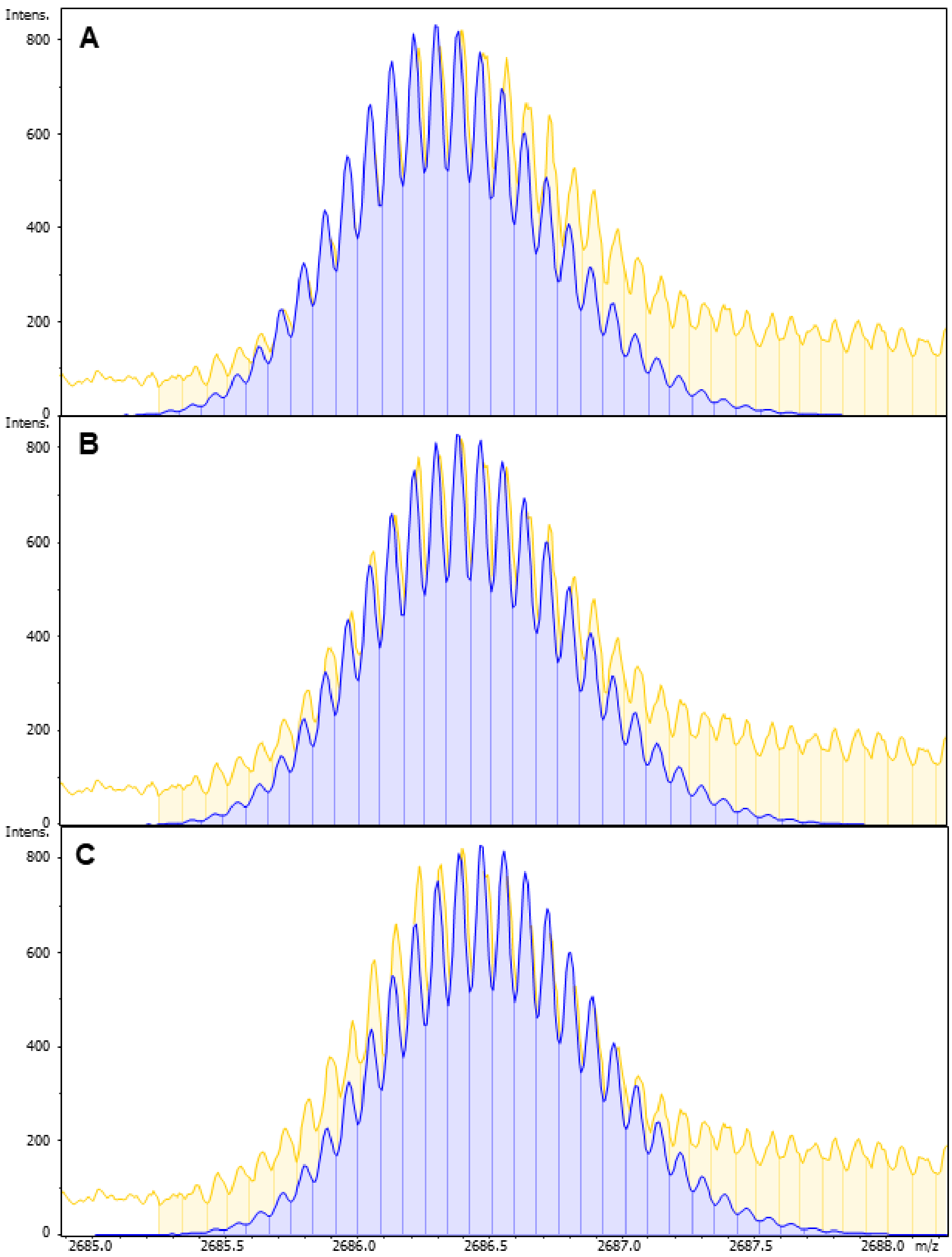

Fig. S9 Overlay of measured (yellow) isotopic pattern of the charge state 12+ of the αβh2-dimer in peak 8 with different simulated isotopic patterns of the 12+ charge state of αβh2-dimer (M) in blue: (A) $[M+\mathbf{10H}]^{12+}$; (B) $[M+\mathbf{11H}]^{12+}$; (C) $[M+\mathbf{12H}]^{12+}$

Supplementary Tables

Table S1 List of all adalimumab proteoforms detected by CZE-MS analysis. Only proteoforms detected in all triplicate experiments are reported. Species are listed for each peak as numbered in Figure S2 in order of increasing mass. Experimental averages mass, standard deviation, Δm and average relative intensity were calculated as average on triplicate experiments. The average relative intensity is calculated based on the average intensity relative to the most abundant proteoform (here G0F/G0F in peak 3). Theoretical masses were calculated using the NIST Mass and Fragment Calculator. Masses of unknown modifications are caluclated between Experimental Average of the G0F/G0F (variant) and Experimental Average of the G0F/G0F (main)

Peak	Proposed primary sequence modification	Proposed N-glycan	Exp. Average Mass	Standard Deviation	Theor. mass	Δm (Da)	Average relative intensity [%]
F1	LC + HC[E1-H228]	-	47680.9	0.3	47680.9	0.0	1.4
F2	LC + HC[Pyr1-D225]	-	47296.3	0.2	47296.5	-0.1	0.5
1	C-terminal proline amidation (both HC)	G0F/G0	147821.7	1.3	147818.3	3.3	0.5
		G0F/G1F -HexNAc	147924.1	2.9	147923.4	0.6	0.6
		G0F/G0F	147967.7	0.2	147964.5	3.2	2.6
		G0F/G1F	148129.6	0.5	148126.6	3.0	1.9
		G1F/G1F	148292.4	0.4	148288.8	3.6	1.5
		G1F/G2F	148455.5	1.9	148450.9	4.6	0.9
		G2F/G2F	148619.4	2.7	148613.0	6.4	0.6
2	C-terminal proline amidation (both HC)	G0F/G1F	148131.2	1.9	148126.6	4.5	0.7
	2 Lys	G0F/G0	148191.9	2.2	148190.8	1.1	0.6
	C-terminal proline amidation (both HC)	G1F/G1F	148295.0	3.3	148288.8	6.2	0.8
	2 Lys	G0F/G0F	148339.2	0.3	148336.9	2.3	1.4
		G0F/G1F	148501.5	0.3	148499.0	2.5	0.7
		G1F/G1F	148663.3	3.1	148661.2	2.1	0.6
3	C-terminal proline amidation (one HC)	G0F/G0	147879.8	0.2	147876.4	3.5	2.7
		G0F/G1F -HexNAc	147984.1	0.3	147981.5	2.7	2.2

		G0F/G0F	148025.3	0.0	148022.5	2.8	15.1
		G0F/G1F	148189.9	1.2	148184.7	5.2	8.8
		G1F/G1F	148350.7	0.6	148346.8	3.9	6.7
		G1F/G2F	148517.9	1.2	148508.9	9.0	4.3
4	C-terminal proline amidation (one HC)	G0F	146577.1	0.8	146577.2	-0.1	0.3
		G1F	146747.0	4.1	146739.3	7.6	0.3
		G0F/G0F	148025.8	0.2	148022.5	3.2	4.8
	1 Lys	G0/G0F	148063.4	0.8	148062.6	0.8	3.4
		G0F/G0F	148210.7	0.5	148208.7	1.9	14.4
		G0F/G1F	148371.4	0.7	148370.9	0.6	6.1
		G1F/G1F	148531.6	2.1	148533.0	-1.4	4.8
		G1F/G2F	148688.1	2.9	148695.2	-7.1	2.2
		G2F/G2F	148855.0	3.0	148857.3	-2.3	1.5
5	Unknown modification of ~+68.9 Da	G0F/G0F	148151.8	0.8	-	-	1.6
		G0F/G1F	148312.1	0.9	-	-	0.9
6	Succinimide	G0/G0F	147918.9	0.2	147917.4	1.5	1.3
		G0F/G1F -HexNAc	148025.2	0.7	148022.5	2.7	1.6
		G0F/G0F	148065.3	0.1	148063.6	1.8	7.6
		G1F/G1F -HexNAc	148186.3	2.3	148184.6	1.7	1.1
		G0F/G1F	148227.3	0.2	148225.7	1.6	3.4
		G1F/G1F	148390.4	1.2	148387.8	2.6	2.7
		G1F/G2F	148560.1	1.5	148550.0	10.2	1.4
		G2F/G2F	148719.2	4.1	148712.1	7.1	1.0
7	N-terminal Asp loss	G0/G0F	147823.5	0.4	147819.3	4.2	0.7
		G0F/G1F -HexNAc	147926.5	0.3	147924.4	2.1	0.8
		G0F/G0F	147968.1	0.1	147965.5	2.6	3.9
		G0F/G1F	148130.2	0.4	148127.6	2.6	2.0
		G1F/G1F	148292.3	0.4	148289.7	2.6	1.7
	Proline amidation on one heavy chain	G0F/G1F +NANA	148475.0	5.5	148475.9	-0.9	1.1
		G1F/G1F +NANA	148638.8	3.7	148638.1	0.8	0.9

		G1F/G2F +NANA	148800.9	1.5	148800.2	0.7	0.8
8	Unknown modification of ~-15.2	G0/G0F	147923.0	0.4	-	-	1.1
		G0F/G1F -HexNAc	148026.3	1.0	-	-	1.9
		G0F/G0F	148067.7	0.7	-	-	6.1
		G0F/G1F	148231.0	0.4	-	-	3.0
		G1F/G1F	148394.8	3.3	-	-	2.8
		G1F/G2F	148560.2	1.7	-	-	1.6
9	Unknown modification of ~42.3 Da	G0/G0F	147893.8	0.8	-	-	1.2
	Main	G0/G0F	147937.0	0.5	147934.4	2.6	1.8
	Unknown modification of ~42.3 Da	G0F/G1F -HexNAc	148000.9	0.8	-	-	1.7
		G0F/G0F	148040.6	0.4	-	-	7.0
	Main	G0F/G0F	148082.0	0.1	148080.6	1.4	8.4
	Unknown modification of ~42.3 Da	G0F/G1F	148202.5	1.3	-	-	4.1
	Main	G0F/G1F	148244.8	0.1	148242.7	2.1	5.3
	Unknown modification of ~42.3 Da	G1F/G1F	148368.1	0.5	-	-	3.4
	Main	G1F/G1F	148407.4	1.1	148404.8	2.6	4.0
		G1F/G2F	148572.1	1.3	148567.0	5.1	2.2
		G2F/G2F	148734.6	0.6	148729.1	5.5	1.4
10	Main	G0F	146637.7	0.1	146635.2	2.5	1.2
		G1F	146799.9	0.3	146797.4	2.5	0.4
		G2F	146963.8	2.1	146959.5	4.3	0.3
		M5/M5	147627.1	0.1	147624.1	3.0	1.0
		G0/G0	147734.5	0.5	147731.2	3.2	1.2
		G0F/G0	147937.4	0.1	147934.4	3.0	15.0
		G0F/G1F -HexNAc	148043.1	0.5	148039.5	3.6	17.1
		G0F/G0F	148082.9	0.1	148080.6	2.3	100.0
		G1F/G1F -HexNAc	148203.9	0.8	148201.6	2.2	10.8
		G0F/G1F	148245.3	0.1	148242.7	2.6	46.6
		G1F/G1F	148407.6	0.4	148404.8	2.7	32.3
		G1F/G2F	148572.8	0.1	148567.0	5.9	13.3

		G2F/G2F	148737.0	1.4	148729.1	7.8	7.7
11	1x Deamidation	G0F	146640.3	2.4	146636.2	4.1	0.3
		G0F/G0	147939.1	0.4	147935.4	3.7	1.5
		G0F/G1F -HexNAc	148047.8	1.6	148040.5	7.3	1.9
		G0F/G0F	148084.9	0.5	148081.5	3.4	9.2
		G0F/G1F	148250.7	0.6	148243.7	7.0	6.2
		G1F/G1F	148413.0	0.8	148405.8	7.1	4.7
	Main	G0F/G1F +NANA	148538.2	1.1	148534.0	4.3	3.5
	1x Deamidation	G1F/G2F	148577.0	2.2	148568.0	9.0	3.1
	Main	G1F/G1F +NANA	148701.1	2.5	148696.1	5.0	2.8
	1x Deamidation	G2F/G2F	148738.0	2.5	148730.1	7.9	2.0
	Main	G1F/G2F +NANA	148862.6	0.4	148858.2	4.3	1.9
		G2F/G2F +NANA	149026.2	2.4	149020.4	5.8	1.0
		G2F/G2F +NANA +Hex	149192.0	1.4	149182.5	9.5	0.6
12	1x Deamidation	G0F	146639.2	1.1	146636.2	3.0	0.3
		G1F	146807.0	1.3	146798.3	8.7	0.2
		G2F	146967.6	4.5	146960.5	7.2	0.2
		G0F/G0	147938.8	0.3	147935.4	3.4	1.3
		G0F/G1F -HexNAc?	148046.6	1.8	148040.5	6.1	1.9
		G0F/G0F	148084.8	0.5	148081.5	3.3	7.8
	Unknown modification of ~+170.3 Da	G0F/G0F	148253.6	0.4	-	-	8.2
		G0F/G1F	148416.1	0.5	-	-	5.8
	Main	G0F/G1F+NANA	148536.6	0.5	148534.0	2.7	7.7
	Unknown modification of ~+170.3 Da	G1F/G1F	148575.9	1.1	-	-	4.5
	Main	G1F/G2F +NANA -HexNAc	148654.6	2.1	148655.0	-0.4	2.4
		G1F/G1F +NANA	148698.6	0.6	148696.1	2.5	5.4
		G1F/G2F +NANA	148861.2	0.2	148858.2	2.9	3.5
		G2F/G2F +NANA	149025.7	0.6	149020.4	5.3	1.6
		G2F/G2F +NANA +Hex	149191.9	2.6	149182.5	9.4	0.9

		G2F/G2F +NANA +2Hex	149357.0	5.3	149344.7	12.3	0.5
13	1x Deamidation	G0F	146638.8	1.3	146636.2	2.6	0.2
		G1F	146801.0	4.9	146798.3	2.7	0.2
		G0F/G0	147938.8	0.2	147935.4	3.4	1.2
		G0F/G1F -HexNAc	148044.6	1.4	148040.5	4.1	1.7
		G0F/G0F	148084.6	0.2	148081.5	3.0	7.7
	Overlap between single deamidated Glycoforms and Glycoforms with a primary sequence modification of ~+171 Da		148249.8	0.4	-	-	6.0
			148301.4	1.9	-	-	2.6
			148412.1	0.3	-	-	4.4
			148576.6	0.7	-	-	2.7
			148740.0	1.1	-	-	1.6
14	1x Deamidation	G0/G0F	147938.6	0.7	147935.4	3.2	1.0
		G0F/G1F -HexNAc?	148045.1	0.4	148040.5	4.6	1.5
		G0F/G0F	148084.7	0.1	148081.5	3.2	6.8
	Overlap between single deamidated Glycoforms and Glycoforms with Glycation		148203.6	0.3	-	-	1.8
			148245.9	0.3	-	-	5.3
			148408.0	0.9	-	-	3.7
			148571.6	1.5	-	-	2.1
			148735.2	1.0	-	-	1.1
	Main	G1F/G1F +2NANA	148990.1	0.4	148987.3	2.7	2.4
		G1F/G2F +2NANA	149152.0	0.3	149149.5	2.5	2.2
		G2F/G2F +2NANA	149313.4	0.3	149311.6	1.8	1.0
		G2F/G2F +2NANA +Hex	149479.9	0.3	149473.8	6.1	0.6
		G2F/G2F +2NANA +2Hex	149646.4	0.8	149635.9	10.5	0.3
		G0F/G0F/G1FS	149983.0	0.9	149979.3	3.7	0.4
		G0F/G1F/G1FS	150145.0	1.1	150141.4	3.5	0.3
		G1F/G1F/G1FS	150303.1	2.3	150303.6	-0.4	0.2
15	Glycation	G0F/G0F	148246.4	2.2	148242.7	3.7	1.9
		G0F/G1F	148409.1	1.1	148404.8	4.3	1.5
		G1F/G1F	148574.3	7.2	148567.0	7.4	1.2

Table S2 List of all infliximab proteoforms detected by CZE-MS analysis. Only proteoforms detected in all triplicate experiments are reported. Species are listed for each peak as numbered in Figure S4 in order of increasing mass. Experimental averages mass, standard deviation, Δm and average relative intensity were calculated as average on triplicate experiments. The average relative intensity is calculated based on the average intensity relative to the most abundant proteoform (here G0F/G0F in peak 1). Theoretical masses were calculated using the NIST Mass and Fragment Calculator

Peak	Proposed primary sequence modification	Proposed N-glycan	Exp. Average Mas	Standard Deviation	Theor. mass	Δm (Da)	Average relative intensity [%]
1	2 Lys	G0F	147328.1	0.1	147323.5	4.5	1.0
		G1F	147487.4	2.5	147485.7	1.7	0.8
		M5/M5	148315.4	0.4	148312.4	3.0	3.0
		G0/G0F -HexNAc	148422.4	0.4	148419.6	2.8	2.4
		G0F/G0F -HexNAc	148570.4	1.0	148565.7	4.7	8.2
		G0/G0F	148626.5	0.3	148622.7	3.7	7.7
		G0F/G1F -HexNAc	148728.7	0.4	148727.8	0.9	15.2
		G0F/G0F	148771.2	0.1	148768.9	2.3	100.0
		G1F/G1F -HexNAc	148888.8	0.8	148890.0	-1.2	12.3
		G0F/G1F	148933.4	0.2	148931.0	2.4	76.9
		G1F/G1F	149095.4	0.2	149093.2	2.3	44.8
		G1F/G2F	149258.5	0.6	149255.3	3.2	18.8
		G2F/G2F	149422.8	0.4	149417.5	5.4	8.2
2	2 Lys	G0F/G0F	148770.8	0.8	148768.9	1.9	2.4
		G0F/G1F	148932.3	1.6	148931.0	1.2	2.0
		G1F/G1F	149094.3	1.9	149093.2	1.2	1.5
		G0F/G1F +NGNA	149240.7	1.2	149238.3	2.4	2.3
		G1F/G1F +NGNA	149402.2	0.8	149400.4	1.8	2.7
		G1F/G2F +NGNA	149564.9	1.5	149562.6	2.4	2.3
		G2F/G2F +NGNA	149727.2	2.4	149724.7	2.5	1.4
3	2 Lys	G1F-HexNAc/G1FS-HexNAc	148994.8	0.2	148994.0	0.8	2.0

		G0F/G1FS-HexNAc	149038.6	0.4	149035.1	3.5	4.2
		G1F/G1FS-HexNAc	149201.5	0.7	149197.2	4.3	4.3
		G2F/G1FS-HexNAc	149364.7	0.9	149359.4	5.3	2.9
		G1F/G1F +NGNA	149400.0	1.7	149400.4	-0.4	1.8
		G2F/G1FS-HexNAc +Hex	149525.7	0.8	149521.5	4.2	1.8
		G1F/G2F +NGNA	149563.5	1.2	149562.6	1.0	1.2
		G2F/G2F +NGNA	149725.0	0.4	149724.7	0.3	0.9
4	2 Lys + 1x Deamidation	G0/G0F	148628.1	1.9	148623.7	4.4	1.3
		G0F/G1F -HexNAc	148728.7	1.2	148728.8	-0.2	1.5
		G0F/G0F	148773.0	0.5	148769.9	3.1	6.0
		G1F/G1F -HexNAc	148891.6	2.0	148891.0	0.7	1.5
		G0F/G1F	148935.0	0.2	148932.0	3.0	4.8
		G1F/G1F	149096.9	0.7	149094.2	2.8	3.2
		G1F/G2F	149259.7	1.1	149256.3	3.4	1.7
		G2F/G2F	149425.8	1.4	149418.4	7.3	1.1
5	1 Lys	G0F	147199.3	0.9	147195.4	3.9	0.8
		G1F	147359.5	2.1	147357.5	2.0	0.6
		M5/M5	148186.8	0.2	148184.2	2.6	1.3
		G0/G0F - HexNAc	148294.3	0.8	148291.4	2.9	1.5
		G0F/G0F - HexNAc	148442.5	0.9	148437.5	5.0	4.7
		G0/G0F	148498.1	0.5	148494.6	3.5	5.0
		G0F/G1F -HexNAc	148600.0	0.2	148599.7	0.4	9.8
		G0F/G0F	148643.3	0.2	148640.7	2.5	67.2
		G1F/G1F -HexNAc	148763.4	0.9	148761.8	1.6	9.7
		G0F/G1F	148805.2	0.1	148802.9	2.3	55.2
		G1F/G1F	148967.2	0.1	148965.0	2.2	32.7
		G1F/G2F	149130.6	0.3	149127.1	3.5	13.6
		G2F/G2F	149295.3	0.4	149289.3	6.0	6.5
6	1 Lys	G0F/G1F +NGNA	149111.6	0.5	149110.1	1.5	1.6
		G1F/G1F +NGNA	149274.1	1.3	149272.3	1.8	1.9

		G1F/G2F +NGNA	149438.4	2.2	149434.4	4.0	1.6
		G2F/G2F +NGNA	149597.4	2.3	149596.5	0.8	1.0
		G2F/G2F +Hex +NGNA	149762.9	6.0	149758.7	4.2	0.8
7	1 Lys	G1F-HexNAc/G1FS-HexNAc	148864.7	1.7	148865.9	-1.1	1.3
		G0F/G1FS-HexNAc	148909.7	0.4	148906.9	2.8	2.7
		G1F/G1FS-HexNAc	149073.3	1.1	149069.1	4.3	2.8
		G2F/G1FS-HexNAc	149236.3	0.6	149231.2	5.1	2.0
		G2F/G1FS-HexNAc +Hex	149396.7	0.9	149393.3	3.4	1.3
8	1 Lys + 1x Deamidation	G0F/G1F -HexNAc	148599.4	1.7	148600.6	-1.2	1.2
		G0F/G0F	148644.5	0.7	148641.7	2.9	3.5
		G0F/G1F	148806.5	0.6	148803.8	2.7	3.0
		G1F/G1F	148969.6	1.8	148966.0	3.6	2.0
		G1F/G2F	149132.8	1.9	149128.1	4.7	1.3
9	0 Lys	G0F	147068.4	1.9	147067.2	1.2	0.7
		G1F	147230.7	1.4	147229.3	1.4	0.5
		M5/M5	148059.5	0.7	148056.0	3.5	1.5
		G0/G0F - HexNAc	148166.1	0.5	148163.2	2.9	1.7
		G0F/G0F - HexNAc	148314.1	0.4	148309.3	4.7	5.9
		G0/G0F	148369.8	0.5	148366.4	3.4	5.1
		G0F/G1F -HexNAc	148471.9	0.4	148471.5	0.4	11.3
		G0F/G0F	148515.0	0.1	148512.5	2.4	76.9
		G1F/G1F -HexNAc	148634.1	0.5	148633.6	0.5	10.5
		G0F/G1F	148677.0	0.2	148674.7	2.3	62.5
		G1F/G1F	148839.1	0.1	148836.8	2.3	37.9
		G1F/G2F	149002.0	0.4	148999.0	3.0	15.9
		G2F/G2F	149166.7	1.1	149161.1	5.6	7.4
		G2F/G2F +Hex	149330.9	0.5	149323.2	7.7	3.2
10	0 Lys	G0F/G1F +NGNA	148984.3	0.2	148981.9	2.4	1.9
		G1F/G1F +NGNA	149146.2	0.3	149144.1	2.1	2.4
		G1F/G2F +NGNA	149308.7	0.0	149306.2	2.5	2.0

		G2F/G2F +NGNA	149470.6	1.3	149468.4	2.2	1.2
		G2F/G2F +Hex +NGNA	149633.7	3.5	149630.5	3.2	0.8
11	0 Lys	G1F-HexNAc/G1FS-HexNAc	148737.6	0.5	148737.7	-0.1	1.4
		G0F/G1FS-HexNAc	148782.2	0.5	148778.7	3.4	3.3
		G1F/G1FS-HexNAc	148945.9	0.5	148940.9	5.0	3.4
		G0F/G1F +NGNA	148981.1	1.4	148981.9	-0.8	1.6
		G2F/G1FS-HexNAc	149108.7	1.7	149103.0	5.7	2.2
		G1F/G1F +NGNA	149142.0	1.8	149144.1	-2.0	1.6
		G2F/G1FS-HexNAc +Hex	149270.3	0.9	149265.2	5.1	1.5
		G1F/G2F +NGNA	149308.2	1.1	149306.2	2.0	1.1
		G2F/G2F +NGNA	149468.1	2.1	149468.4	-0.3	0.9
12	1x Deamidation	G0F/G0F	148516.6	0.9	148513.5	3.1	3.0
		G0F/G1F	148678.0	0.5	148675.7	2.3	2.5
		G1F/G1F	148840.2	0.7	148837.8	2.4	1.8
		G1F/G2F	149001.0	1.2	148999.9	1.1	1.0
13	1x Deamidation	G0F/G0F	148516.2	0.2	148513.5	2.6	2.2
		G0F/G1F	148678.1	0.2	148675.7	2.4	1.9
		G1F/G1F	148840.7	1.1	148837.8	2.9	1.4
		G1F/G2F	149003.9	2.5	148999.9	3.9	1.0
14	1x Deamidation	G0F/G0F	148515.9	0.7	148513.5	2.3	3.0
		G0F/G1F	148678.1	0.4	148675.7	2.4	2.6
		G1F/G1F	148840.7	0.1	148837.8	2.9	1.7
		G1F/G2F	149004.6	0.8	148999.9	4.7	1.0

Table S3 List of all bevacizumab proteoforms detected by CZE-MS analysis. Only proteoforms detected in all triplicate experiments are reported. Species are listed for each peak as numbered in Figure S5 in order of increasing mass. Experimental averages mass, standard deviation, Δm and average relative intensity were calculated as average on triplicate experiments. The average relative intensity is calculated based on the average intensity relative to the most abundant proteoform (here G0F/G0F in peak 3). Theoretical masses were calculated using the NIST Mass and Fragment Calculator

Peak	Proposed primary sequence modification	Proposed N-glycan	Exp. Average Mass	Standard Deviation	Theor. Mass	Δm (Da)	Average relative intensity [%]
F1	LC + HC[E1-H230]	-	48207.1	0.92	48,207.33	-0.3	0.6
F2	HC + HC[S106-G452] + 2 LC	G0F	136097.8	0.01	136096.3	1.5	0.2
		G0F/G0F -HexNAc	137342.1	0.47	137338.5	3.7	0.3
		G0F/G1F -HexNAc	137498.6	0.33	137500.6	-2.0	0.6
		G0F/G0F	137544.3	0.21	137541.7	2.6	9.1
		G0F/G1F	137706.8	0.04	137703.8	3.0	2.0
		G1F/G1F	137868.8	0.18	137865.9	2.8	1.5
		G1F/G2F	138034.8	1.11	138028.1	6.8	0.5
		G2F/G2F	138195.8	4.16	138190.2	5.6	0.2
1	1 Lys	G0F/G0	149183.6	1.04	149179.6	4.0	1.3
		G1F/G0F -HexNAc	149279.8	0.86	149284.7	-4.9	1.4
		G0F/G0F	149328.3	0.08	149325.7	2.5	20.9
		G1F/G0F	149490.6	0.24	149487.9	2.7	4.4
		G1F/G1F	149652.7	0.82	149650.0	2.6	3.7
		G1F/G2F	149818.9	0.99	149812.2	6.7	1.1
		G2F/G2F	149983.6	5.22	149974.3	9.3	0.6
		G2F/G2F +Hex	150146.6	1.04	150136.4	10.2	0.2
2	1 Lys	G0F	147884.0	2.65	147880.4	3.6	0.5
		G1F	148040.4	16.29	148042.5	-2.2	0.4

		G2F	148213.8	6.65	148204.7	9.2	0.3
		G0F/G0F -HexNAc	149125.8	2.18	149122.5	3.3	0.6
		G0F/G0F	149325.3	6.69	149325.7	-0.4	1.1
		G0F/G1F	149484.7	8.89	149487.9	-3.2	0.9
		G1F/G1F	149651.5	4.44	149650.0	1.5	0.7
		G1F/G2F	149811.7	1.47	149812.2	-0.5	0.4
		G2F/G2F	149962.8	23.24	149974.3	-11.5	0.3
3	Succinimide	G0F/G0F	149182.3	0.17	149182.3	0.0	2.1
		G1F/G0F	149341.2	0.85	149341.2	0.0	0.6
		G1F/G1F	149508.0	7.21	149508.0	0.0	0.5
		G1F/G2F	149668.3	12.88	149668.3	0.0	0.2
4	Main	G0F -HexNAc	147551.8	0.15	147549.0	2.8	0.3
		G0	147611.0	0.81	147606.1	4.9	0.2
		G1F -HexNAc	147709.2	0.30	147,711.18	-2.0	0.4
		G0F	147755.1	0.19	147752.23	2.8	4.1
		G1F	147917.0	0.15	147914.4	2.7	1.0
		G2F	148078.6	0.42	148076.5	2.1	0.7
		G2F +Hex	148246.2	0.97	148238.7	7.5	0.2
		G0F/G0F -2HexNAc	148793.4	0.06	148791.2	2.2	0.9
		G0F/G0 -HexNAc	148850.6	0.50	148848.2	2.4	0.2
		G0F/G0F -HexNAc	148997.4	0.28	148994.4	3.1	7.1
		G0F/G0	149055.9	0.21	149051.4	4.4	4.5
		G0F/G1F -HexNAc	149154.4	0.17	149156.5	-2.1	8.7
		G0F/G0F	149200.0	0.10	149197.6	2.5	100.0
		G0F/G1F	149362.4	0.26	149359.7	2.7	23.5
		G1F/G1F	149524.5	0.33	149521.9	2.6	18.0
		G1F/G2F	149691.7	0.33	149684.0	7.7	5.6
		G2F/G2F	149853.6	1.47	149846.1	7.5	3.1
		G2F/G2F +Hex	150017.6	0.40	150008.3	9.3	1.1

5	1x Deamidation	G0F	147756.8	0.34	147752.2	4.5	0.8
		G1F	147917.2	0.86	147914.4	2.8	0.3
		G2F	148081.3	1.88	148076.5	4.8	0.2
		G0F/G0F -HexNAc	149000.8	1.15	148995.4	5.5	0.1
		G0/G0F	149060.9	2.80	149052.4	8.5	0.3
		G0F/G0F	149201.6	0.31	149198.6	3.0	7.5
		G0F/G1F	149363.8	0.84	149360.7	3.1	2.2
		G0F/G2F	149526.4	0.44	149522.8	3.6	1.7
		G1F/G2F	149695.0	1.85	149685.0	10.0	0.6
		G2F/G2F	149854.8	4.66	149847.1	7.7	0.3
6	1x Deamidation	G0F	147757.0	1.18	147752.2	4.7	0.6
		G1F	147916.1	4.92	147914.4	1.7	0.3
		G2F	148084.1	6.04	148076.5	7.6	0.2
		G0F/G0F -HexNAc	148999.2	0.75	148995.4	3.8	0.4
		G0/G0F	149057.8	1.01	149052.4	5.4	0.4
		G0F/G1F -HexNAc	149156.3	0.30	149157.5	-1.2	0.9
		G0F/G0F	149201.5	0.28	149198.6	3.0	10.5
		G0/G0F +HexNAc	149258.2	0.90	149,255.6	2.6	2.0
		G0F/G1F	149363.9	0.32	149360.7	3.2	3.3
		G0F/G2F	149526.2	0.71	149522.8	3.3	2.5
		G1F/G2F	149692.4	0.73	149685.0	7.4	0.9
		G2F/G2F	149855.7	1.50	149847.1	8.6	0.4
8	1x Deamidation	G0F	147756.0	1.02	147752.2	3.8	0.6
		G1F	147917.2	2.25	147913.3	3.9	0.3
		G0F/G0F -HexNAc	148999.2	0.86	148995.4	3.9	0.4
		G0/G0F	149057.4	0.97	149052.4	5.0	0.3
		G0F/G1F -HexNAc	149155.9	0.75	149157.5	-1.6	0.9
		G0F/G0F	149201.6	0.13	149198.6	3.0	10.2
		G0/G0F +HexNAc	149263.9	0.30	149,255.61	8.3	1.2

		G0F/G1F	149363.6	0.34	149360.7	2.9	3.2
		G0F/G2F	149526.2	0.95	149522.8	3.4	2.3
		G1F/G2F	149692.7	1.52	149685.0	7.7	0.9
		G2F/G2F	149856.5	2.35	149847.1	9.4	0.4
		G2F/G2F +Hex	150018.3	3.81	150009.3	9.0	0.2
